# Carbon Accumulation in Tropical Dry Forests and Savannas Despite Widespread Land‐Cover Change, Revealed by Radar Remote Sensing

**DOI:** 10.1111/gcb.71141

**Published:** 2026-10-19

**Authors:** Sam B. Harrison, John L. Godlee, David T. Milodowski, Reza Asiyabi, Penelope J. Mograbi, Zhofre Aguirre, Jarcilene S. Almeida, George K. D. Ametsitsi, Alejandro Araujo‐Murakami, Sally Archibald, Luzmila Arroyo Padilla, Lorena Benitez, Samuel J. Bowers, Thomas K. Brade, Domingos Cardoso, João M. B. Carreiras, Antonio Alberto Jorge Farias Castro, Duncan Chalo, Halidou Compaore, Isabela S. C. da Costa, Ítalo Antônio Cotta Coutinho, Eder C. das Neves, Vera De Cauwer, Tony César de Sousa Oliveira, Hermane Diesse, Tomas F. Domingues, Fernando Elias, Camila Laís Farrapo, G. Feig, Ted R. Feldpausch, Moabe F. Fernandes, David R. Galbraith, Nestor I. Gasparri, Emanuel Gloor, Francisco Maiato, Tatenda Gotore, Karollyne S. Guimarães, Gloria Gutierrez‐Sibauty, Rhett D. Harrison, Thomas P. Higginbottom, Francoise Yoko Ishida, Hamza Issifu, John Ngugi Kigomo, Timothy J. Killeen, Aurora Levesley, Simon L. Lewis, José Romualdo S. Lima, Dante Loto, Elisvan Mariano Sousa, Beatriz S. Marimon, Ben Hur Marimon Junior, Mário Marcos do Espírito Santo, Antonia M. L. Marques, Matheus Santos Martins, Collins W. Masinde, Musingo Tito E. Mbuvi, Iain M. McNicol, Karina Melgaço, B. Mogonong, Peter W. Moonlight, Paulo S. Morandi, Leonor Patricia Cerdeira Morellato, Magna Soelma Beserra de Moura, Anderson Muchawona, Jonathan Ilunga Muledi, Leena Naftal, Jhonathan Oliveira Silva, Catherine L. Parr, Nayane Cristina C. S. Prestes, Luciano Paganucci de Queiroz, Tshililo Ramaswiela, Desirée M. Ramos, Simone Matias Reis, Natasha S. Ribeiro, Raphael R. Rocha, Priscyla Maria Silva Rodrigues, Gustavo Saiz, Rubens M. Santos, Mylor Ngoy Shutcha, Domingos Fortunato P. F. da Silva, Kathleen G. Smart, Bonaventure Sonké, Elmar Veenendaal, Chemuku Wekesa, Lucy H. Wells, Débora Zuanny, Timothy R. Baker, Kyle G. Dexter, Gabriele C. Hegerl, R. Toby Pennington, Oliver L. Phillips, Stephen Sitch, Ann Carla Staver, Mathew Williams, Shaun Quegan, Steven Hancock, Nicole Helene Augustin, Casey M. Ryan

**Affiliations:** ^1^ School of GeoSciences University of Edinburgh Edinburgh UK; ^2^ Forest Global Earth Observatory Smithsonian Tropical Research Institute Washington DC USA; ^3^ Carrera de Ingeniería Forestal Universidad Nacional de Loja Loja Ecuador; ^4^ Departamento de Botânica, Centro de Biociências Universidade Federal de Pernambuco Recife Pernambuco Brazil; ^5^ CSIR‐Forestry Research Institute of Ghana Kumasi Ghana; ^6^ Instituto de Investigaciones Forestales de la Amazonía, Universidad Autónoma del Beni José Ballivián (IIFA‐UAB) Bolivia; ^7^ Museo de Historia Natural Noel Kempff Mercado Universidad Autónoma Gabriel René Moreno (UAGRM) Santa Cruz de la Sierra Bolivia; ^8^ School of Animal, Plant and Environmental Sciences University of the Witwatersrand Johannesburg South Africa; ^9^ Universidad Autónoma Gabriel René Moreno Museo de Historia Natural Noel Kempff Mercado Santa Cruz Bolivia; ^10^ Jardim Botânico Do Rio de Janeiro (JBRJ) Rio de Janeiro RJ Brazil; ^11^ Departamento de Biologia (Aposentado) da Universidade Federal do Piauí Brazil; ^12^ Department of Biology, Faculty of Science and Technology University of Nairobi Nairobi Kenya; ^13^ Centre National de la Recherche Scientifique et Technologique Institut de L'environnement et de Recherches Agricoles Ouagadougou Burkina Faso; ^14^ Programa de Pós‑Graduação em Sistemática, Uso e Conservação da Biodiversidade (PPGSis) Universidade Federal do Ceará (UFC) Fortaleza CE Brazil; ^15^ Universidade do Estado de Mato Grosso (UNEMAT) Nova Xavantina, Mato Grosso Brazil; ^16^ Biodiversity Research Centre Namibia University of Science and Technology Windhoek Namibia; ^17^ Ongava Research Centre Namibia; ^18^ Institute of Biogeosciences IBG2: Plant Sciences, Forschungszentrum Jülich GmbH Jülich Germany; ^19^ Faculty of Communication and Environment Hochschule Rhein‐Waal Kamp‐Lintfort Germany; ^20^ Namibia University of Science and Technology Windhoek Namibia; ^21^ Departamento de Biologia, Faculdade de Filosofia, Ciências e Letras de Ribeirão Preto Universidade de São Paulo (FFCLRP‐USP) Ribeirão Preto Brazil; ^22^ Museu Paraense Emílio Goeldi Belém Brazil; ^23^ Universidade Federal de Lavras (UFLA) Lavras Minas Gerais Brazil; ^24^ Department of Geography, Geoinformatics and Meteorology University of Pretoria Hatfield South Africa; ^25^ South African Environmental Observation Network (SAEON) South Africa; ^26^ Faculty of Environment, Science and Economy University of Exeter Exeter UK; ^27^ Royal Botanic Gardens Kew, Surrey UK; ^28^ School of Geography University of Leeds Leeds UK; ^29^ Instituto de Ecología Regional Universidad Nacional de Tucumán‐ CONICET Argentina; ^30^ Universidade Mandume Ya Ndemufayo Lubango Angola; ^31^ Herbário do Lubango ISCED‑Huíla (Instituto Superior de Ciências da Educação da Huíla) Lubango Angola; ^32^ cE3c – Centro de Ecologia, Evolução e Alterações Ambientais, Faculdade de Ciências Universidade de Lisboa Campo Grande, Lisboa Portugal; ^33^ Santa Cruz, Asociación Boliviana Para la Conservación de Las Aves Santa Cruz de la Sierra Bolivia; ^34^ CIFOR‐ICRAF Lusaka Zambia; ^35^ Airbus Defence and Space, Surrey Research Park Guildford UK; ^36^ Centre for Tropical Environmental and Sustainability Science, and College of Science and Engineering James Cook University Cairns Queensland Australia; ^37^ Department of Forestry & Forest Resources Management, Faculty of Natural Resources & Environment University for Development Studies Tamale Ghana; ^38^ Kenya Forestry Research Institute (KEFRI) Nairobi Kenya; ^39^ Missouri Botanical Garden St Louis Missouri USA; ^40^ Department of Geography University College London UK; ^41^ Federal University of Agreste of Pernambuco Garanhuns Pernambuco Brazil; ^42^ Instituto de Silvicultura y Manejo de Bosques (INSIMA), Consejo Nacional de Investigaciones Científicas y Técnicas (CONICET), Universidad Nacional de Santiago del Estero Santiago del Estero Argentina; ^43^ Universidade Estadual de Montes Claros, UNIMONTES Montes Claros Brazil; ^44^ Universidade de Brasília Brazil; ^45^ BeZero Carbon UK; ^46^ Healthy Ecosystems, Institute of Natural Resources Pietermaritzburg South Africa; ^47^ Botany, Trinity College Dublin Dublin 2 Ireland; ^48^ Center for Research on Biodiversity Dynamics and Climate Change and Department of Biodiversity, Phenology Lab Biosciences Institute, UNESP—São Paulo State University Rio Claro, São Paulo Brazil; ^49^ Brazilian Agricultural Research Corporation (Embrapa) Embrapa Tropical Agroindustry Fortaleza CE Brazil; ^50^ Zimbabwe Forestry Commission Zimbabwe; ^51^ Faculté Des Sciences Agronomiques Université de Lubumbashi Democratic Republic of the Congo; ^52^ Colegiado de Ecologia Universidade Federal do Vale do São Francisco (UNIVASF) Senhor do Bonfim Brazil; ^53^ School of Environmental Sciences University of Liverpool Liverpool UK; ^54^ Department of Zoology & Entomology University of Pretoria Pretoria South Africa; ^55^ Departamento de Ciências Biológicas Universidade Estadual de Feira de Santana (UEFS) Feira de Santana BA Brazil; ^56^ Instituto Tecnológico Vale Belém Pará Brazil; ^57^ Centro de Ciências Biológicas e da Natureza Universidade Federal do Acre Rio Branco Acre Brazil; ^58^ Laboratório de Ecologia de Ecossistemas Florestais e Savânicos Universidade Federal do Acre Rio Branco Acre Brazil; ^59^ Department of Forest Engineering Eduardo Mondlane University Maputo Mozambique; ^60^ Universidade Estadual de Feira de Santana (UEFS) Feira de Santana BA Brazil; ^61^ Department of Environment and Agronomy Instituto Nacional de Investigación y Tecnología Agraria y Alimentaria, Consejo Superior de Investigaciones Científicas (INIA‐CSIC) Madrid Spain; ^62^ Avenida Allioun Blondy Beye, bairro Académico Instituto Superior de Ciências de Educação do Huambo, ISCED‐Huambo Huambo Angola; ^63^ Plant Systematics and Ecology Laboratory, Higher Teachers' Training College University of Yaoundé I Yaoundé Cameroon; ^64^ Plant Ecology & Nature Conservation Group Wageningen University Wageningen the Netherlands; ^65^ Kenya Forestry Research Institute Taita Taveta Research Centre Wundanyi Kenya; ^66^ School of Biological Sciences University of Edinburgh Edinburgh UK; ^67^ Royal Botanic Garden Edinburgh Edinburgh UK; ^68^ Department of Life Sciences and Systems Biology University of Turin Turin Italy; ^69^ Geography, University of Exeter UK; ^70^ Department of Biology Washington University St Louis Missouri USA; ^71^ Ecology and Evolutionary Biology Princeton University USA; ^72^ National Centre for Earth Observation UK; ^73^ University of Sheffield Sheffield UK; ^74^ School of Mathematics University of Edinburgh Edinburgh UK

**Keywords:** aboveground biomass, carbon stocks and fluxes, deforestation, degradation, land‐use change, savannas, synthetic aperture radar, tropical dry forests, woody carbon, woody encroachment

## Abstract

Accurate mapping of woody biomass and its temporal change is critical for understanding the global carbon balance, informing restoration and supporting evidence‐based policy on climate mitigation and livelihoods. The vegetation of the dry tropics, comprised largely of dry forests and savannas, holds substantial woody carbon stocks, but both stocks and change over time remain uncertain. Where Earth Observation (EO) data have been used to estimate change over time, different products give conflicting results on the direction, magnitude and location of change. We present pan‐dry‐tropical maps of aboveground carbon density (AGCD) and AGCD change at 100 m resolution for the years 2015–2024, developed using ALOS ScanSAR satellite radar observations calibrated with in situ measurements (*n* = 237). These maps suggest an aboveground woody carbon pool (AGC) in the dry tropics of 25.3 ± 2.0 PgC, ~13.0% of global AGC. Net AGC has increased by 0.43 ± 0.07 PgC yr^−1^, ~20.0% of the global net land sink. The overall net increase (0.43 PgC yr^−1^) is comprised of gross losses from deforestation and degradation (0.27 ± 0.05 PgC yr^−1^) and gains due to densification and extensification of 0.59 ± 0.1 PgC yr^−1^, with the balance accounted for by small changes (change < 20% of AGCD; 0.11 PgC yr^−1^). Losses are equally split between degradation and deforestation, while gains are divided roughly 2:1 between the densification of woody ecosystems and expansion into previously grass‐dominated areas. These new maps, created with a dry‐tropics‐specific EO method, highlight the importance of tropical dry forests and savannas in the global carbon cycle. The prominent role of degradation‐related losses suggests that climate change mitigation will need to focus much more on reducing losses from overharvesting than at present. The widespread increases in carbon density and the increase in tree biomass into open systems present major challenges for biodiversity conservation, restoration and livelihoods.

## Graphical abstract

We map aboveground carbon density (AGCD) across tropical dry forests and savannas at 100 m for 2015–2024 using ALOS‑2 L‑band radar calibrated with field plots. Aboveground carbon (AGC) stocks total 25.3 ± 2 PgC (13% of global AGC) and net AGC is increasing at 0.43 ± 0.07 PgC yr^−1^ (20% of the land sink). Gains reflect densification and extensification, and losses are split evenly between degradation and deforestation.

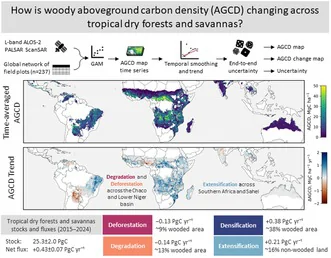

## 1 Introduction

Together, tropical dry forests and savannas are the predominant vegetation of the tropics, supporting 2 billion livelihoods (Pennington et al. 2018; Ryan et al. 2016) and a unique biodiversity (Abreu et al. 2017; Pennington et al. 2018; Portillo‐Quintero et al. 2015). These biomes are undergoing rapid change, driven by new land uses (Rudel 2017; Schröder et al. 2021), resource extraction (Arnan et al. 2018; Wessels et al. 2013), agricultural expansion (Curtis et al. 2018), altered fire regimes (Andela et al. 2017), and climate change, such as changing rainfall frequency and intensity (IPCC 2023; Siyum 2020).

Despite pressures that reduce woody biomass, there is evidence that areas of dry forests and savannas function as a significant and increasing carbon sink (Ahlström et al. 2015; Higgins and Scheiter 2012; Sitch et al. 2015). The vegetation of the dry tropics is thought to hold substantial woody carbon stocks (Hunka et al. 2025; Santoro 2018) but both stocks (Hunka et al. 2025), and especially changes over time (Ernst et al. 2024), remain highly uncertain, partly due to a lack of high‐quality field reference data for the calibration and validation of remote sensing methods. As a result, the size and location of the putative carbon sink remain controversial (Bar‐On et al. 2025; Pan et al. 2024; Randerson et al. 2025).

Dry forests and savannas are undergoing both gains and losses of aboveground biomass, and the net balance remains unclear. Loss processes include a reduction in aboveground woody carbon area‐density (AGCD, MgC ha^−1^) due to overharvesting (commonly for wood fuels or timber; Ryan et al. 2016) or land use changes, primarily conversion to agriculture (Wells et al. 2022). However, alongside livelihood activities that drive loss processes, humans also plant trees and manage land, both actively and passively, to increase woody biomass for various reasons, including the contribution trees make to local livelihoods (Zomer et al. 2016) and as part of conservation and carbon offset projects (Agarwal et al. 2025).

Processes driving AGC gains include enhanced growth or reduced mortality of woody plants, resulting in encroachment (here divided into increased woody density and the expansion of woody plants into previously open/grassy ecosystems). While both processes increase the AGCD, they may well lead to different socio‐ecological impacts. For example, an increase in the area of land dominated by woody species, often termed ‘woody plant encroachment’ or ‘woody thickening’, leads to the loss of open habitat, loss of water availability, and reduced availability of grazing land for livestock (García Criado et al. 2020; Skhosana et al. 2025; Stevens et al. 2017). The processes driving these gains remain contested and are likely regionally specific (Venter et al. 2018), with candidate mechanisms including CO_2_ fertilisation (Archer et al. 2017), the suppression of fire (Andela et al. 2017), the loss of mega‐herbivores (Stevens et al. 2017) but also overgrazing by domestic animals (Sankaran et al. 2008) and climatic changes that favour trees over grass (Archer et al. 2017; Stevens et al. 2017).

These gain and loss processes lead to a complex mosaic of changes across landscapes, often with high heterogeneity at fine resolution. For example, gains due to fire suppression often occur in close proximity to losses due to human activities, including small‐scale agriculture (McNicol et al. 2018, 2023; Ryan et al. 2012). This fine‐scale variability has made it hard to quantify these processes with coarse‐resolution remote sensing (Boitard et al. 2025; Dou et al. 2023; Li et al. 2025; Wigneron et al. 2024) and has led to the underestimation of carbon losses due to land management (Arneth et al. 2017). The lack of accurate fine‐scale data on carbon stock changes has also limited our ability to understand the spatial pattern and relative importance of the assumed drivers of change.

As a result of this data gap, efforts to manage these biomes have been based on weak evidence. For example, tackling deforestation has been a focal point of efforts to mitigate land use emissions, often under Reducing Emissions from Deforestation and Forest Degradation (REDD+) programmes, but recent work has suggested that ‘degradation’ (in this context, defined as a reduction in AGCD, without conversion to another land use) may be a more important source of emissions (Gao et al. 2020; McNicol et al. 2023; Pearson et al. 2017; Ryan et al. 2012). (Note that despite the terminology, reductions in AGCD are not synonymous with degradation in the sense of a loss of ecosystem services.) Similarly, there is widespread concern about the biodiversity and livelihood implications of woody encroachment (Stevens et al. 2017), but limited systematic data on which biomes, regions and social contexts are most affected. The data gap has also hindered the development of sustainable wood fuel policies and harvesting regimes (Ghilardi et al. 2018) because wood extraction is often considered to be unsustainable even in the absence of data on sustainable harvesting rates (Wells et al. 2022). Despite smallholders often being blamed for reductions in tree biomass (Curtis et al. 2018; Kissinger et al. 2012; Pendrill et al. 2022), it may be that these activities are holding in check woody expansion into open ecosystems due to climate change or CO_2_ fertilisation. Without robust data at relevant spatial resolutions on the extent and rate of these land‐cover changes, and the associated carbon fluxes, none of these debates can be resolved.

Accurate mapping of biomass and its temporal changes is thus critical for many policy domains (Cook‐Patton et al. 2021; Herold et al. 2019; Luvuno et al. 2018; Pan et al. 2024). While there has been progress in AGCD mapping using a range of remote sensing technologies, these are often focused on moist forests, and as yet these have not been able to provide a consistent view of AGCD change in dry forests and savannas at high enough resolution or over sufficient time scales to inform these debates. The most established technologies, utilising passive optical and near‐infrared reflectances (Landsat, MODIS, Sentinel‐2) have struggled to map change over time in these mixed tree‐grass ecosystems (Pletcher et al. 2024), and while they show promise in mapping woody cover, they are not able to systematically retrieve AGCD or its change (Brandt et al. 2016; Hansen et al. 2019; Staver and Hansen 2015). Newer, active sensors, such as the ICESat and GEDI LiDAR missions, have produced AGCD estimates (Duncanson et al. 2022), but suffer from limited spatial coverage (discrete footprints), limited calibration of the relationship between the retrieved metrics and AGCD, and a short observational period. Passive microwave remote sensing has been able to estimate long‐term AGCD change (Boitard et al. 2025; Li et al. 2025; Wigneron et al. 2024), but only at coarse resolution (e.g., L‐VOD datasets typically have a spatial resolution of 25 km; Zhang et al. 2025) and, given the large pixel size, these products are impossible to directly calibrate or validate. Recent progress with high‐resolution optical data mapping individual tree crowns is promising (Brandt et al. 2020), but a lack of a long‐term archive of imagery means it will not be able to deliver information about decadal changes in woody biomass for some time. Various maps have used a fusion of these data sources, often in combination with machine learning approaches, but with ad hoc and limited calibration data (e.g., Rodríguez‐Veiga et al. 2025), and such approaches often are limited by the changing earth observation (EO) data availability over the last decades and a lack of systematic calibration and validation in the tropics (Dion et al. 2024), especially in dry forests and savannas.

Long‐wavelength, space‐borne Synthetic Aperture Radar (SAR) has the potential to overcome some of these challenges for biomass estimation in dry tropical environments. The sensitivity of radar backscatter to vegetation structure makes it particularly valuable for detecting changes in woody structure and biomass over time (Carreiras et al. 2026; Wessels et al. 2023). JAXA's ALOS‐1 and ‐2 missions offer a c. 25 m resolution dataset covering most of the last two decades, and the PALSAR instrument provides L‐band (23 cm wavelength) radar backscatter observations which are strongly related to woody biomass (Brolly and Woodhouse 2014; Gou et al. 2022; Rodríguez‐Veiga et al. 2020; Ryan et al. 2012; Wessels et al. 2023). These data contribute to one of the current leading AGCD datasets (CCI‐Biomass; Santoro and Cartus 2025), which is currently the only multi‐temporal global AGCD product publicly available at sub‐kilometre resolution. However, the CCI‐Biomass dataset has limitations, exhibiting systematic biases and regional inconsistencies when validated against ground‐based estimates or compared with other products (Araza et al. 2023; Hunka et al. 2025). This is particularly evident in the tropics, primarily related to a lack of calibration and validation reference field data, and some data availability and quality issues with the ALOS archive (Araza et al. 2022). As a result of these limitations, the current version of the CCI‐Biomass product flags its estimates of year‐to‐year change as ‘improbable’ in all but 4% of dry tropical pixels. Here we try to address some of the current limitations of AGCD change mapping in tropical savannas and dry forests, using a pan‐dry‐tropics‐specific SAR‐biomass model using L‐band SAR combined with a new, extensive field dataset for calibration and validation. We use the resulting maps to answer the following questions about the aboveground carbon dynamics of these biomes:

1. How much carbon is stored in the aboveground biomass of tropical dry forests and savannas, and what contribution do these biomes make to the global carbon sink?
2. What are the spatial patterns of net biomass change across tropical dry forests and savannas?
3. What are the carbon losses due to deforestation (a change in land use) and how do they compare to losses due to reductions in carbon area‐density (likely due to tree harvesting or excess mortality)?
4. What are the carbon gains associated with densification (increases in woody biomass within existing wooded areas) and extensification (expansion of woody cover into non‐woody areas)?
5. Where are the hotspots of these land‐cover change processes?

## 2 Methods

Our overall approach involved (i) delineating the extent of dry forests and savannas in both transformed and untransformed landscapes; (ii) parametrising a model of the relationship between radar observations and AGCD using (iii) plot data and (iv) ALOS‐2 PALSAR‐2 ScanSAR observations. We then conducted (v) end‐to‐end uncertainty propagation and (vi) model validation. The model is then used to (vii) predict AGCD time series across all dry forests and savannas. We then (viii) use these time series to understand the carbon dynamics associated with the major land‐cover changes.

### 2.1 The Extent of Tropical Dry Forests and Savannas

We study the full biogeographic extent of tropical dry forests and savannas in the year 2020, including land that has been human‐transformed (Section S2). Tropical dry forests are seasonally dry, generally with semi‐deciduous or drought‐deciduous trees and a minimal grass layer. Savannas are primarily also seasonally dry ecosystems but contain a continuous C4 grassy layer and variable open tree cover where the tree–grass balance is often maintained by fire and/or herbivory (Keith et al. 2022).

We delineated vegetated tropical dry forests and savanna lands first by excluding extratropical and very arid areas using climate data, as well as Mediterranean and temperate climates (Muñoz‐Sabater et al. 2021). Within these climate bounds, we exclude vegetation that is not likely dry forest or wooded savannas using land‐cover data by excluding non‐vegetated land covers and evergreen closed‐canopy forest (CGLS‐LC100; Buchhorn et al. 2020, and MCD12Q1 for water bodies). We also use the Terrestrial Ecoregions of the World dataset (Dinerstein et al. 2017) to aid delineation in human‐transformed areas where the current anthropic land covers make it difficult to identify vegetation types. The Ecoregions dataset maps ecosystems of regional extent that share similar environmental conditions and species assemblages (Dinerstein et al. 2017). To integrate across the multiple spatial scales of these land‐cover products, we classify all tropical landscapes as dry forest/savanna and transformed/untransformed at a larger spatial scale (10 × 10 km). This scale preserves meaningful heterogeneity in vegetation type and transformation status, while reducing sensitivity to edge effects and random error in the land‐cover products. It also allows for more accurate classification of mosaic landscapes typical of dry forests and savannas, where woody patches are interspersed with grass‐dominated land covers. Our own uncertainty analysis (Section 3.2) shows how uncertainty in AGCD declines from pixel to multi‐kilometre scales. Our study area was defined as all landscape units where the majority of the landscape unit was classed as dry forest and savanna.

We classified the landscape units into those where the dominant land cover is largely natural (hereafter ‘untransformed’) and those where humans have altered the land cover to another class, such as urban or crop agriculture (hereafter, ‘human‐transformed’). The latter definition does not extend to transformations such as grazing by domesticated animals and the dominance of invasive species, which are extremely hard to map at a global scale. We classify land as transformed if the majority of the landscape unit comprises anthropic land covers (CGLS‐LC100; Buchhorn et al. 2020) or had experienced complete tree cover loss since 2000 (Hansen et al. 2013). Transformed landscapes therefore include large‐scale commercial agriculture, smallholder agricultural systems, mosaics of agriculture among remnant and secondary woody vegetation, and cleared and abandoned land. Untransformed landscapes may still experience significant human pressures (e.g., wood harvesting), but the land cover has not been changed to a predominantly anthropic one. Full details of the extent mapping and transformed landscape delineation are in (Section S2).

Tree plantations are not distinguished from other woody land covers in this study. Plantations are classified as forest by the land cover maps used to distinguish transformed and untransformed land, but at the landscape scale they may still fall into either class as described above. Any growth or rotation of plantation stands is captured by the same land cover change processes applied elsewhere, without distinguishing planted woody vegetation. As a result, some of the AGCD changes observed here reflect the growth and harvesting of plantation crops rather than the recovery, expansion and deforestation of natural woody vegetation. An estimate of the role of plantations is provided in (Section S4), based on the Global Forest Management dataset 2015 (Lesiv et al. 2022). This product maps planted and plantation forest for the year 2015, the start year of this study. We estimate the area of plantations in our domain, as well as the average rate of loss and rate of gain of AGCD in plantations to understand the contribution of plantations to net carbon changes.

### 2.2 Radar‐AGCD Model

To quantify the relationship between woody biomass and radar backscatter we use field‐estimated AGCD as the response variable and radar backscatter as the predictor, using a generalised additive model (GAM; Wood 2017). GAMs provide a flexible, nonlinear modelling approach, which is required because the functional form of the biomass‐backscatter relationship impacts AGCD retrieval and is contested (Joshi et al. 2017; Mermoz et al. 2015; Mitchard et al. 2009; Ryan et al. 2012; Wessels et al. 2023). While it is well known that the relationship saturates at higher biomass, it is unclear at what level (Brolly and Woodhouse 2014; Joshi et al. 2017); a GAM allows this saturation to be defined by the data.

To parametrise the model we use AGCD estimated at field plots (see Section 2.3) and spatially coincident observations of the normalised radar cross section ($\gamma^{0}$, m^2^ m^−2^) for the same year from JAXA's ALOS‐2 PALSAR‐2 sensor, in ScanSAR mode (see Section 2.4). For each plot (*p*), we use the annual minimum of the radar observations in the year (*t*) of the most recent field data collection ($\min\left(\gamma_{p,t}^{0}\right)$). The biomass‐backscatter relationship is therefore parametrised using data drawn from diverse years.

The final model is a GAM with a Gamma distribution and log link and takes the form:

(1) $$\log_{e}\left(E\left(\mathrm{AGC}D_{p,t}\right)\right)=\alpha +f\left(\min\left(\gamma_{p,t}^{0}\right)\right)$$

where $\mathrm{AGC}D_{p,t}=E\left(\mathrm{AGC}D_{p,t}\right)+\epsilon_{p,t}$, with $\epsilon_{p,t}∼N\left(0,\sigma^{2}\right).$

$E\left(\mathrm{AGC}D_{p,t}\right)$ is the expected AGCD, α is the intercept, $\epsilon_{p,t}$ is the random error, and the nonlinear one‐dimensional smooth function $f\left(\right)$ has a cubic regression spline basis for the backscatter predictor variable and 6 dimensions of the basis function. Weights were applied based on the plot size (square root of plot size in hectares).

We checked the model by examining partial effect plots, evaluating residuals for normality and heteroskedasticity, and checking for spatial autocorrelation by interpreting the semi‐variogram of the residuals. We also checked that the dimension of the basis was sufficient (Section S5). The modeling was conducted in R using the mgcv package (S.Wood 2000).

### 2.3 Field Estimates of AGCD

We use 237 large (≥ 0.25 ha) research plots across the global extent of tropical dry forests and savannas to provide reference data for calibration of the model Equation (1) and validation. These plots came from the SECO partnership, consisting of plots from ForestPlots.net (ForestPlots.net et al. 2021; Lopez‐Gonzalez et al. 2011), the SEOSAW Partnership (2021), TERN (Sparrow et al. 2024) via the ausplotsR package (Guerin et al. 2023) and other independent sources. We followed standard biometric methods to estimate aboveground woody biomass and convert it to AGCD (Martin et al. 2018). This includes the use of a refined stem biomass allometry specific to dry forests and savannas (Godlee et al. in review). The plots are circular, square or rectangular with an area ≥ 0.25 ha, to ensure coverage by at least four 25 m‐SAR pixels. We omitted plots on steep slopes (> 20°) as these cause problems for radar imaging (foreshortening and layover). We used plots that have been censused during the ALOS‐2 ScanSAR observation period (2015–2024). The uncertainty of plot‐estimated AGCD was quantified using a Monte Carlo procedure that accounted for errors in biometry, allometry and wood density, detailed in (Section S3.1) uncertainty. The plots are grouped across 98 sites, which are geographical clusters of plots established by the same research group, distinguished from other sites by the same research group by being > 100 km apart and/or in a different vegetation type.

### 2.4 SAR Data

We used multi‐temporal L‐band SAR data from ALOS‐2 PALSAR‐2 in ScanSAR mode (level 2.2; Shimada et al. 2014) from the years 2015 to 2024. Many previous efforts to map biomass using PALSAR data relied on JAXA's annual mosaic data, but the ScanSAR dataset offers an improvement on the mosaic's annual frequency with acquisitions that cover the entire tropical region roughly every 42 days (JAXA 2023). ScanSAR data are provided with a pixel spacing of 25 m and were processed at this resolution. We applied data quality masks and speckle filtering (refined Lee 3 × 3, Lee et al. 2009) to each image. From all images acquired, we took the minimum pixel value of the HV polarisation in each year for each 25 m pixel and then resampled to 100 m resolution using mean aggregation. Use of the annual minimum helps to minimise the impacts of soil moisture on backscatter (Gao et al. 2021; Lucas et al. 2010), because the minimum backscatter is normally obtained from the driest time of the year in these highly seasonal ecosystems. ScanSAR data availability differs spatially and from year to year, ranging from 0 to 80 images per year when including overlapping scenes (Figure S1.1); as such, further masks were applied to remove pixel‐years with insufficient numbers of observations. PALSAR observations were masked using continent‐specific masking as each continent had different ScanSAR data provision and quality issues (see Section S1.2). Masked pixel‐years were filled either via temporal interpolation (see Section 2.5) or with a spatial gap‐filling method where most data in the time series were missing (see Section S8).

### 2.5 Prediction and Validation of AGCD

The parametrised model Equation (1) was used to predict AGCD across the full extent defined in Section 2.1 for all pixel‐years with suitable PALSAR‐2 observations from 2015 to 2024, giving a time series of AGCD predictions.

We use a holdout cross‐site validation approach to validate model predictions, splitting our dataset into training and testing sets with one site held out. We hold out by site (i.e., a cluster of plots, *n* = 98) rather than just by plot to reduce the effect of spatial autocorrelation on our validation (Ploton et al. 2020). We refitted the model *n* = 98 times (once for each hold‐out site) and used it to make predictions for AGCD for each plot in the holdout site for all years with good radar data. This gives us a time series of AGCD predictions for the holdout plots, and we then carry out multi‐temporal smoothing on these data to gap‐fill and reduce noise. Given the short time series (maximum 10 years), we keep the temporal window size for smoothing at five years. We tested a backward‐looking rolling average and Savitzky–Golay smoothing (Savitzky and Golay 1964), with Savitzky–Golay smoothing chosen as it provided improved validation results. From the temporally smoothed time series across all plots, we selected the prediction for the year of the field data. We performed this holdout procedure for all sites to generate our validation dataset. We report systematic deviation (Equation 2) and root mean squared deviation (RMSD; Equation (3)) for the full validation set following Duncanson et al. (2021). Biomass range‐specific validation is shown in (Section S5.1).

(2) $$\text{Systematic deviation}=\frac{1}{N}\sum_{p=1}^{N}\left({\hat{\text{AGCD}}}_{p,t}-\mathrm{AGC}D_{p,t}\right)$$

(3) $$\text{RMSD}=\sqrt{\frac{1}{N}\sum_{p=1}^{N}{\left({\hat{\text{AGCD}}}_{p,t}-\mathrm{AGC}D_{p,t}\right)}^{2}}$$

${\hat{\text{AGCD}}}_{p,t}$ is the predicted AGCD at 100 m resolution for plot p, in the year of the corresponding plot data census t, using the holdout model, averaging all pixels that intersect the plot boundaries. $\mathrm{AGC}D_{p,t}$ is the field‐estimated AGCD for plot p, in the year of the census t. As both the prediction and the observations of AGCD are uncertain (see below), we also report a comparison of uncertainty distributions in (Section S5.1).

We also compare our holdout predictions against an existing global AGCD product, CCI‐Biomass (v6.0; Santoro and Cartus 2025), using the same validation statistics as above.

### 2.6 Uncertainty Propagation and Attribution

To understand the uncertainty on pixel‐level predictions of AGCD we conducted an end‐to‐end error propagation. We then upscaled the error propagation to understand the uncertainty on zonal statistics (i.e., aggregating multiple pixels) of AGC stock and change (see below). Our estimation of the error on predictions of AGCD ($\sigma_{\hat{\text{AGCD}}}$) accounts for both model error ($\sigma_{\mathrm{GAM}}$) and for the fact that the training and prediction data, plot‐estimated AGCD and $\min\left(\gamma^{0}\right)$, have uncertainties ($\sigma_{\text{AGCD}}$ and $\sigma_{\min\left(\gamma^{0}\right)}$, respectively).

We estimate $\sigma_{\hat{\text{AGCD}}}$ with a Monte Carlo procedure. For each of 10,000 simulations it: (i) draws a value of each plot's $\mathrm{AGC}D_{p}$ from the plot‐specific probability distributions arising from biometric and allometric model uncertainty (defined in Section S3.1); (ii) draws a value of $\min\left(\gamma^{0}\right)$ for each plot from a probability distribution function (pdf), defined given the observed $\min\left(\gamma^{0}\right)$, see below; (iii) fits the GAM defined in Equation (1) (Section 2.2) using the sampled data; and (iv) samples from the posterior parameter distribution of the GAM (sections 5.8 and 6.10 in Wood 2017) to predict AGCD over the range of observed $\min\left(\gamma^{0}\right)$, sampling again from the pdf of $\min\left(\gamma^{0}\right).$

The pdf of $\min\left(\gamma^{0}\right)$ is defined as normally distributed with a mean equal to the observation and a standard deviation of *x*% of the mean. x was quantified from the variation of the minimum backscatter of four overlapping scenes. At 500 random locations in our study domain, we took a random year and found the annual minima for four scenes that overlapped. From the four annual minima we took the standard deviation. Across the 500 locations, the average standard deviation was 12% of the mean.

To quantify the contribution of each source of uncertainty, we repeat this process, excluding all but one source of uncertainty (i.e., only sampling the one source of uncertainty; all other values fixed at their mean) with 3000 simulations for each source.

Our uncertainty analysis does not include the uncertainty from the representativeness of calibration plots relative to the domain, nor does it include errors arising from the definitions we use for land‐cover change processes. These represent structural sources of uncertainty that are difficult to capture statistically and are discussed as limitations of the study.

### 2.7 AGCD Change Mapping

To estimate change over time, we cleaned and temporally smoothed the $\hat{\text{AGCD}}$ time series and then fit a linear regression of change over time for each pixel, using the slope of the regression as the annual rate of change in AGCD ($\Delta \hat{\text{AGCD}}$). This rate of change characterises the decadal trend in AGCD and, although presented as an annualised value, is not intended to reflect year‐to‐year changes.

To understand the carbon dynamics associated with the major land‐cover changes, we classify biomass change into four categories, extending the logic of McNicol et al. (2018).

- ‘Deforestation’—i.e., a loss of biomass likely to correspond to a new land use or clearcutting, is defined as woody carbon loss (> 20% of stocks) where a pixel moves from a wooded to non‐wooded state. The threshold we use to define ‘wooded’ is 10 MgC ha^−1^, which roughly equates to the 10% forest cover definition used by many countries and the United Nations Food and Agricultural Organisation (FAO; McNicol et al. 2018).
- We use ‘Degradation’ to refer to woody systems that undergo AGCD reductions of > 20% and that remain above the wooded threshold at the end of the time series. Note that this is a convenience term used in the sense of the REDD+ terminology and is not intended to reflect any changes in ecosystem services other than aboveground carbon storage.

Often referred to using the terms ‘woody plant encroachment’ or ‘woody thickening’ in the literature, we split gain processes into:

- ‘Densification’: areas that undergo an AGCD increase of > 20% in already‐wooded areas (using the same ‘wooded’ threshold as above), and
- ‘Extensification’: non‐wooded areas that increase in AGCD by > 20% and cross the woody threshold to become wooded.

Losses and gains < 20% of the starting value are recorded as minor losses/gains and not assigned to any of the land‐cover change processes.

### 2.8 Zonal Statistics and Their Uncertainty

We summarise carbon stock, carbon stock change, area affected by each land‐cover change, and the associated carbon dynamics, over multiple zonal domains continents, selected ecoregions and selected countries; in (Section S2). At these larger‐than‐pixel scales, uncertainty estimation depends on the spatial and temporal covariance of the errors on Δ AGCD. To assess how spatial scale influences our uncertainty estimates accounting for multiple uncertainty sources is computationally infeasible for every pixel at pan‐tropical scale with 100 m resolution. Instead, we implemented a Monte Carlo simulation to propagate measurement and model uncertainties to multiple window sizes (100, 25, 10, 1 km, 500 and 100 m) at 156 stratified random locations in our domain (for smaller windows, four replicates were placed in the corners of the 100 km window). Locations were stratified by mean Δ AGCD at the 100 km scale. Following the same approach for uncertainty estimation described above, we conducted 500 Monte Carlo simulations predicting all pixel‐years in the window, with each simulation propagating the sources of uncertainty as above. We again gap‐fill and apply temporal smoothing to each pixel's time series. For each simulation of each window, we estimate the mean AGCD and Δ AGCD and report the percentage standard error as the standard deviation of the 500 simulations as a proportion of the mean. Averaging across the 156 locations allows us to characterise how spatial scale (i.e., window size) influences this uncertainty. When we present zonal statistics for domains larger than the largest window (100 km), we use the 100 km window uncertainties as a conservative estimate of the true uncertainty.

## 3 Results

### 3.1 Radar‐AGCD Model

The plot‐based biomass–radar backscatter relationship for tropical dry forests and savannas shows a broadly linear form up to a median saturation point of around 52.0 MgC ha^−1^ (IQR: 47–60; Figure 1). In 42% of model simulations, there was no saturation (i.e., the model did not estimate a slope ≤ 0). The GAM explained 56% of the deviance and passed the checks for basis dimension, normality and the lack of spatial pattern in the residuals (Section S5).

**FIGURE 1:**
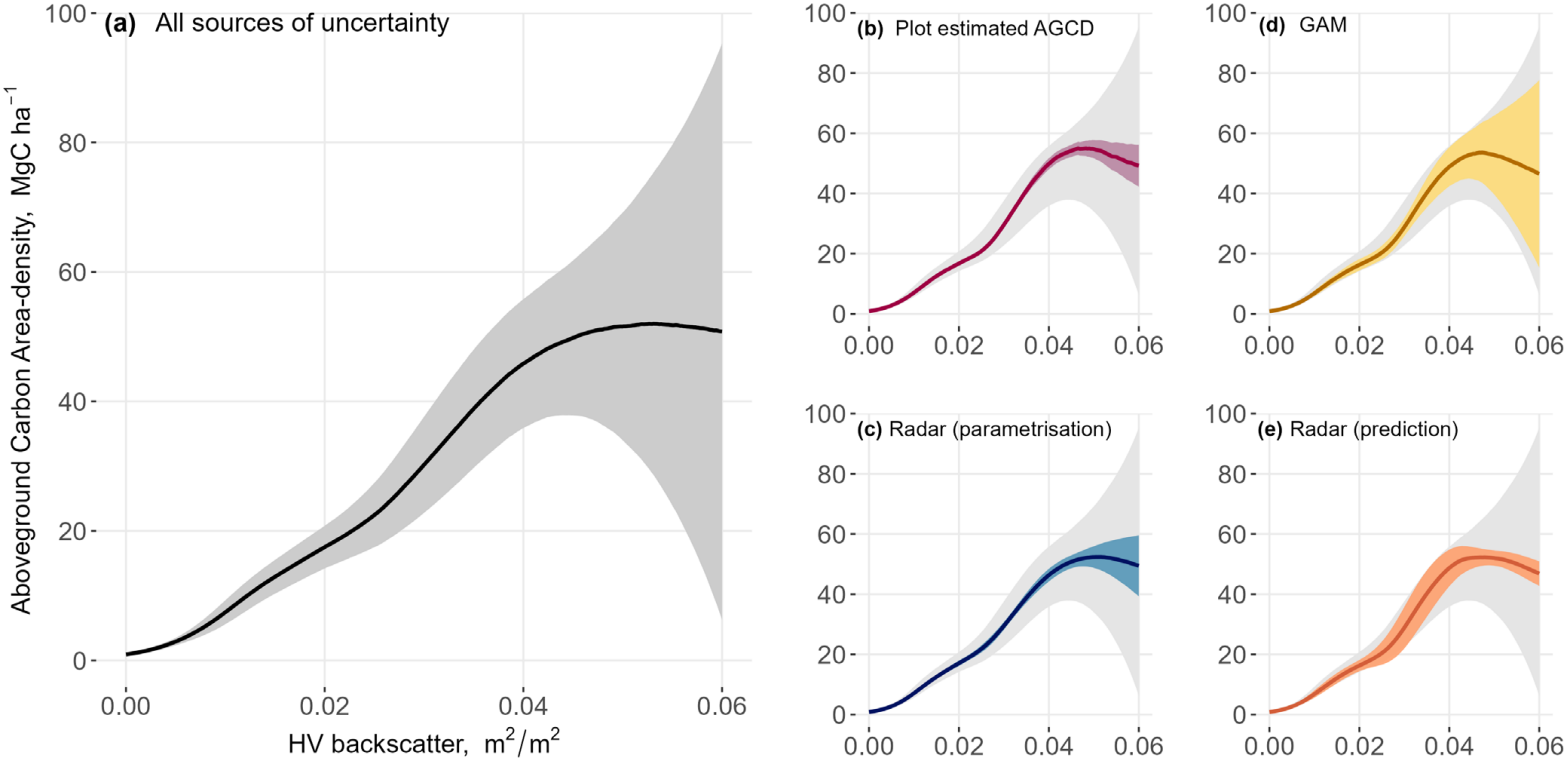
The biomass–radar backscatter relationship for tropical dry forests and savannas (a). Solid lines show the mean and the shaded area shows the standard deviation of predictions from the Monte Carlo error propagation simulations. Grey shading (all panels) shows the full uncertainty on AGCD predictions which is caused by uncertainty in: (b) plot estimates of AGCD used for model parametrisation ($\sigma_{\text{AGCD}}$); (c) the radar data used to parametrise the model ($\sigma_{\min\left(\gamma^{0}\right)}$); (d) model fitting and prediction ($\sigma_{\mathrm{GAM}}$), and (e) the radar data used to predict AGCD (orange). The model is fitted with data from 237 plots (see Section S3).

The standard deviation of the predictions was around 15% of the mean for much of the AGCD range (Figure 1), but increased in proportional terms at both low and high values of $\min\left(\gamma^{0}\right)$ (Section S5.2). This uncertainty in prediction of AGCD stems mostly from model uncertainty and from uncertainty in the radar data used for prediction. To illustrate the contribution of each source of uncertainty, at the pan‐tropical mean AGCD of 12.3 MgC ha^−1^, the uncertainty in the radar data used for prediction were 68% of the full uncertainty, while model error was 54% (note that these are not additive). The proportional contributions are similar (73% and 54%) at 5 MgC ha^−1^, which is considered a lower bound for AGCD retrieval using L‐band data (McNicol et al. 2018). At the upper bound of AGCD sensitivity (~50 MgC ha^−1^) model uncertainty dominates (56%) with the contribution from the radar data used for prediction dropping to 46% of total uncertainty.

Using our leave‐one‐site‐out holdout procedure for cross‐validation, we find that model predictions are moderately accurate, with a plot‐scale RMSD of 15.1 MgC ha^−1^ and a systematic deviation of 6.1 MgC ha^−1^ (Figure 2). Predictions were more accurate for lower biomass plots (see Section S5.1), with overpredictions at high biomass. Continent‐ and biome‐specific validation shows spatial variation in RMSD, likely due to sample size differences in the reference data and structural and allometric heterogeneity among biomes (Section S5.1). In comparison, the CCI‐Biomass product has a worse RMSD of 19.8 MgC ha^−1^, and although it has a lower overall systematic deviation (0.6 MgC ha^−1^), it has less sensitivity, with underestimation at low values (at AGCD values < 10 MgC ha^−1^, CCI systematic deviation = −4.5 MgC ha^−1^ versus this study = −0.8 MgC ha^−1^) and overestimation at high values at AGCD values > 50 MgC ha^−1^, 42.7 vs. 24.2; (Section S6).

**FIGURE 2:**
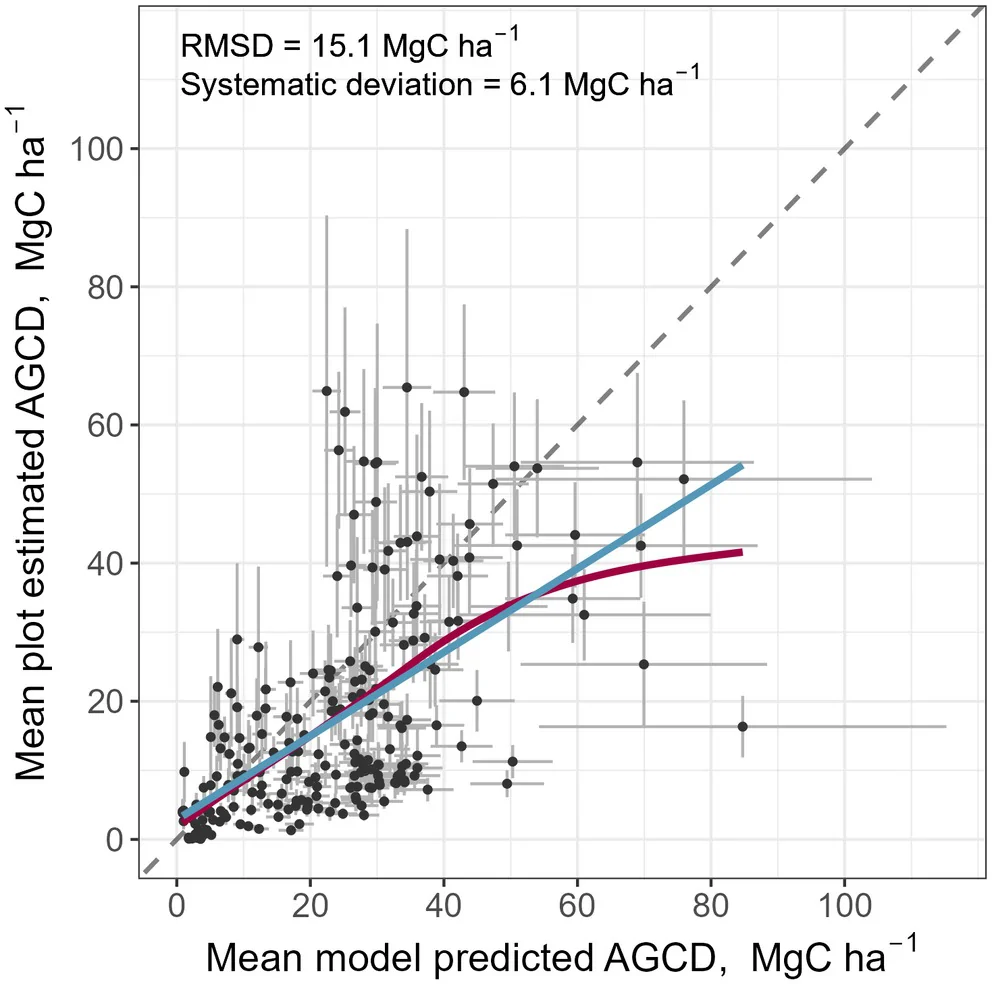
Validation of the AGCD‐backscatter model using holdout sites. Points show the field‐estimated AGCD and the model prediction (mean of the Monte Carlo simulations). Lines show ± one standard deviation of the simulations. The dashed line indicates 1:1; the thick red line shows a GAM smooth, and the blue line shows the linear fit. RMSD = Root Mean Squared Deviation. The model predictions are based on a leave‐one‐site‐out procedure and the uncertainties are estimated through a Monte Carlo simulation that considers multiple sources of uncertainty on the field‐measured AGCD as well as errors in the radar data and model uncertainty.

As both the reference data (ground measured AGCD) and model predictions have uncertainty, we compared their distributions. On 60.3% of plots, there was an overlap between the 95th percentile confidence intervals of the two estimates; on 30.5% of plots, the standard errors overlapped.

### 3.2 Zonal Uncertainties

To understand how these plot/pixel‐scale errors combine over larger areas, we ran Monte Carlo simulations at 6 scales, at 156 random locations. Zonal mean AGCD had uncertainties that decreased with scale, with the standard deviation of the simulations decreasing from 15% of the mean at the pixel scale to 8% at 100 × 100 km (Table 1). AGCD change showed a similar pattern, but with higher uncertainties, decreasing from 37% at the pixel scale to 17% at 100 × 100 km (Table 1).

**TABLE 1:** Uncertainties in AGCD and AGCD change vary with spatial scale.

Zone window size (km)	Number of zones	AGCD uncertainty (std. dev. as % of mean)	AGCD change uncertainty (std. dev. as % of mean)
0.1 × 0.1	526	15	37
0.5 × 0.5	566	12	26
1 × 1	566	11	24
10 × 10	569	10	19
25 × 25	600	9	18
100 × 100	156	8	17

*Note:* The standard deviation of the predicted values as a percentage of the mean is shown at each scale (mean for each window size). At 156 random locations across the dry tropics we used 500 Monte Carlo simulations to propagate the errors in the input data and model through to predictions of AGCD and its change over time over zones of different window sizes. For the smaller window sizes, four replicates were placed in each corner of the 100 × 100 window. Zones in the smaller widow size were excluded if they were not fully in the study domain, leading to *n* < 624.

### 3.3 Pan‐Tropical Carbon Stocks and Dynamics

In total, dry forests and savannas cover 20.5 million km^2^ (Figure 3). The mean AGCD averaged across the study time period and domain is 12.3 ± 1.0 MgC ha^−1^, with total AGC stocks of 25.3 ± 2.0 PgC. In comparison, CCI‐Biomass and GEDI's Level 4B gridded biomass product estimate the stock at 23.2 PgC and 15.6 PgC, respectively. While our stock estimates roughly match those from CCI‐Biomass, there are significant spatial differences between these products (S 6; note that a comparable error estimate for these zonal statistics is not available). Two‐thirds of the dry forest and savanna carbon stock is in Africa (16.7 ± 1.3 PgC over 12.2 M km^2^), followed by the tropical Americas (6.7 ± 0.5 PgC, 4.5 M km^2^) and Asia and Australia (2.0 ± 0.2 PgC, 3.7 M km^2^). African untransformed landscapes alone hold over half the AGC stock of dry forests and savannas.

**FIGURE 3:**
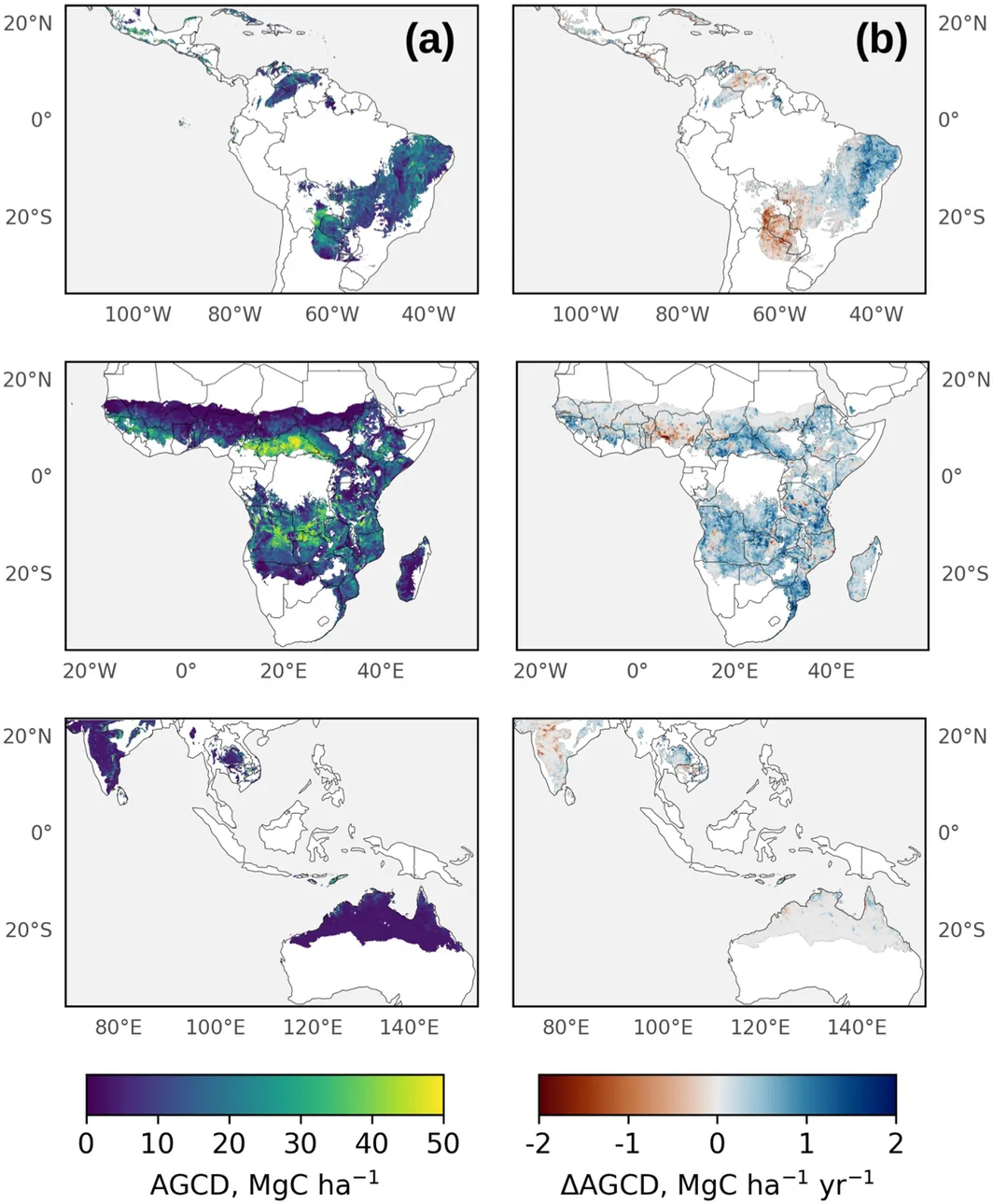
(a) Map of pan‐tropical aboveground carbon density (AGCD) for dry forests and savannas for the decade 2015–2024 (temporal mean). AGCD is predicted from a GAM using ground data and ALOS ScanSAR data and (b) map of AGCD change per year (linear trend) for dry forests and savannas 2015–2024. Both maps have been gap‐filled using a second model based on the ALOS PALSAR mosaic data (see Section S8). Map lines delineate study areas and do not necessarily depict accepted national boundaries.

Of our full domain, in 2020 roughly a third of the landscape units met our criteria for transformed land, i.e., the landscape unit was mostly crops, had been deforested between 2000 and 2020, or comprised another anthropic land cover. This 7.36 M km^2^ of transformed land holds ~25% of the AGC in dry forests and savannas, averaging 6.9 ± 0.6 PgC over 2015–2024, with a landscape unit mean AGCD (10.6 ± 0.9 MgC ha^−1^) only slightly lower than the pan‐dry‐tropical mean. The regional proportion of the transformation of land area ranges from 95% of the study domain in Asia, 44% in the tropical Americas, and 33% in Africa to < 1% in Australia. It should be noted that grazed land or areas transformed by exotics do not meet our criteria for transformation, due to the challenges of mapping these land use transformations (Oliveira et al. 2020).

Net AGC change (Figure 3) was positive over the study period (2015–2024) with a total of 0.43 ± 0.07 PgC yr^−1^ (1.7% yr^−1^ of the time‐average stock). While there were net gains across all continents, this varied widely from 2.1% yr^−1^ across Africa, 0.9% yr^−1^ in the tropical Americas and 0.5% yr^−1^ in Asia and Australia. This means that 83% of the increase in stocks is in Africa (0.36 ± 0.06 PgC yr^−1^) compared to only 14% in the Americas (0.06 PgC yr^−1^). Across our domain, the rate of net accumulation of AGC was twice as fast in untransformed land (Table 2), suggesting that transformation greatly impairs the carbon sink function of these biomes.

**TABLE 2:** Zonal statistics of the carbon dynamics of the dry forests and savannas, for the full study extent and broken down by transformed/untransformed status.

	Dry forests and savannas		
	Full extent	Untransformed	Transformed
**(a) AGC (PgC)**	25.34	18.44	6.90
**(b) ΔAGC (PgC yr^−1^)**	0.43	0.36	0.07
Deforestation	−0.13	−0.05	−0.07
Degradation	−0.14	−0.08	−0.05
Densification	0.38	0.29	0.09
Extensification	0.21	0.13	0.08
**(c) ΔAGC (% of stock yr^−1^)**	1.68	1.94	0.98
Deforestation	−0.50	−0.29	−1.05
Degradation	−0.54	−0.46	−0.78
Densification	1.51	1.59	1.29
Extensification	0.83	0.71	1.14
**(d) Area (M km^2^)**	20.54	13.18	7.36
Deforestation	0.76	0.36	0.40
Degradation	1.03	0.65	0.38
Densification	3.11	2.39	0.73
Extensification	1.82	1.20	0.62
**(e) Area (% of initial land cover)**			
Deforestation	9	6	18
Degradation	13	11	17
Densification	38	40	32
Extensification	16	18	13

*Note:* (b and c) Show the AGCD change associated with each of the land‐cover change processes, and (d and e) report the area affected by these processes. Values shown for (a and b) are the totals for our domain, averaged over the time series (2015–2024). (d and e) Show the area affected by each land‐cover change, summed over the time series. Uncertainties on stocks are likely < 8% of the totals and 17% of the changes.

The net change of 0.43 PgC yr^−1^ masks significant spatial variability in regions exhibiting large gains and losses, which comprise gains of +0.59 ± 0.1 PgC yr^−1^ and losses of −0.27 ± 0.05 PgC yr^−1^, with the balance being accounted for by minor changes of low intensity (+0.11 ± 0.02 PgC yr^−1^; Table 2).

### 3.4 Regional Variability in Carbon Dynamics

Carbon stocks are heterogeneously distributed across dry forests and savannas (Figure 3a). Our models predict high biomass on the northern forest‐savanna fringe of the Congo Basin (mean AGCD 34.3 ± 2.7 MgC ha^−1^), as well as the fringes of West African moist forests and across the Miombo woodlands of southern Africa (mean AGCD 19.9 ± 1.6 MgC ha^−1^; total AGC 4.93 PgC). In South and Central America, the highest AGCD is found in parts of the Caatinga (15.3 ± 1.2 MgC ha^−1^; 1.1 ± 0.03 PgC), Mexican dry forests, and the Chaco (17.1 ± 1.4 MgC ha^−1^; 1.1 ± 0.03 PgC). In comparison, the mean AGCD in the Cerrado is 13.3 ± 1.1 MgC ha^−1^; 2.3 ± 0.07 PgC. The rest of the dry forest and savanna extent shows lower biomass values, particularly in the more arid regions; however, small pockets of high biomass are present throughout the dry tropics.

The rate of carbon stock change also displays significant spatial heterogeneity (Figure 3b), with broadly distributed gains punctuated by hotspots of losses that are primarily attributed to land‐cover change (Figure 4). Rapid net increases in AGC are underway in the Caatinga (4.4% yr^−1^), the Miombo in southern Africa (2.2% yr^−1^), and the Central African Republic (1.7% yr^−1^). Net increases are also observed in mainland Southeast Asia (3.8% yr^−1^), but here the gains are mostly in transformed landscapes and 13.6% of the gains from extensification and densification occur in plantations (Section S4). Marked net losses occur across the Chaco (−2.8% yr^−1^) and Nigeria and Benin (−2.1% yr^−1^). There are only small absolute changes underway in Australia, India and the Sahelian savannas (Sudanian and Sahelian Acacia savanna ecoregions), but there are large proportional changes (1%–1.5% yr^−1^) underway in some parts of the Sahelian savannas and the more open savannas of southern Africa (Kalahari woodland and savanna ecoregions; Figure 4).

**FIGURE 4:**
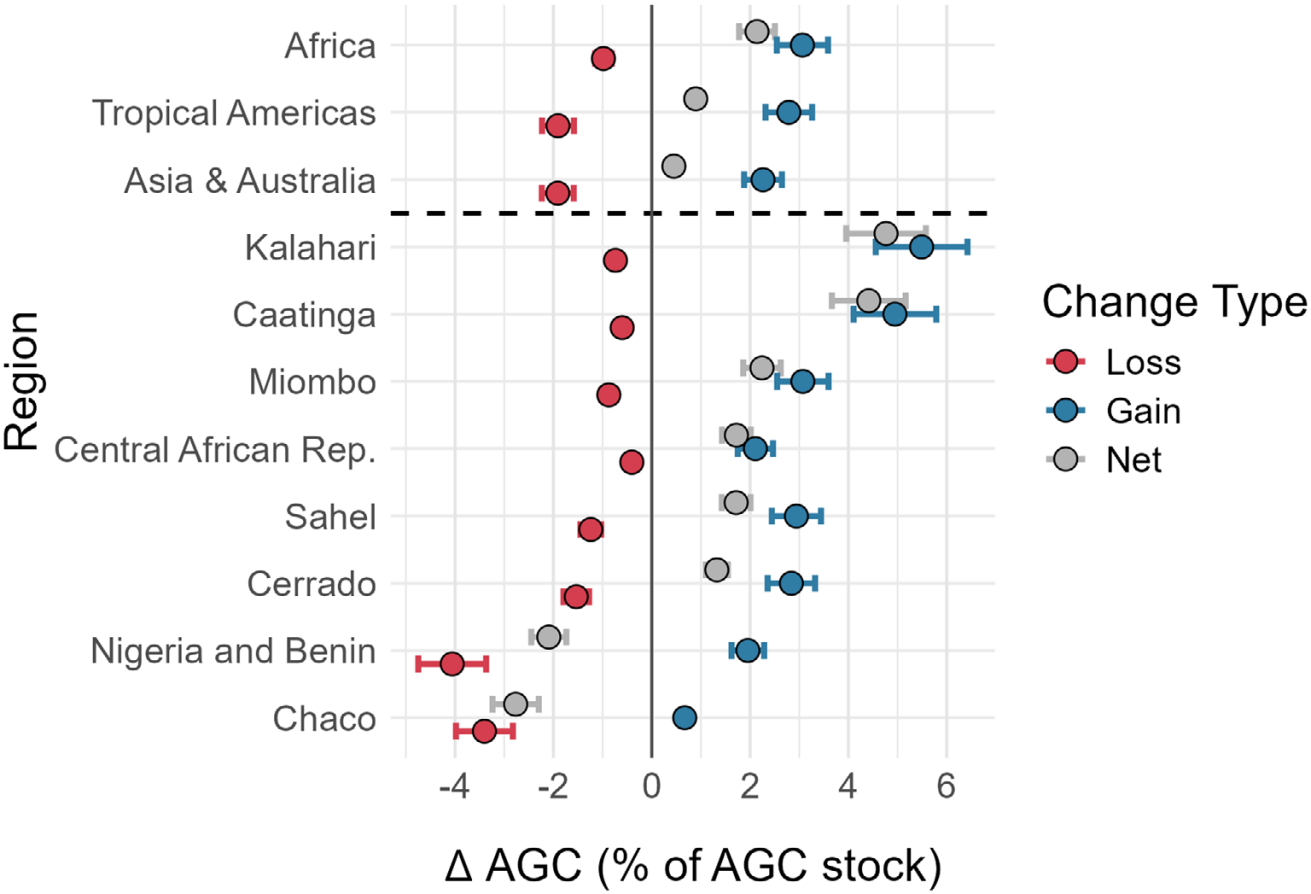
The rate of carbon stock changes in a selection of ecoregions and countries.

### 3.5 Land‐Cover Changes and Emissions

#### 3.5.1 Pan‐Tropical Importance of Different Land‐Cover Change Processes

The losses and gains are driven by both area‐density changes (densification and degradation) and changes in areal extent (extensification and deforestation; Figure 5). For gains, densification (0.38 PgC yr^−1^) was the most extensive process (38% of wooded land) and accounted for 64% of the carbon gains. Extensification was less important in carbon terms (0.21 PgC yr^−1^) but occurred in 16% of non‐woody land. Losses affected 22% of the land area, split roughly evenly in terms of carbon change between deforestation (−0.13 PgC yr^−1^) and degradation (−0.14 PgC yr^−1^), but degradation affected a larger area than deforestation (13% vs. 9% of wooded land).

**FIGURE 5:**
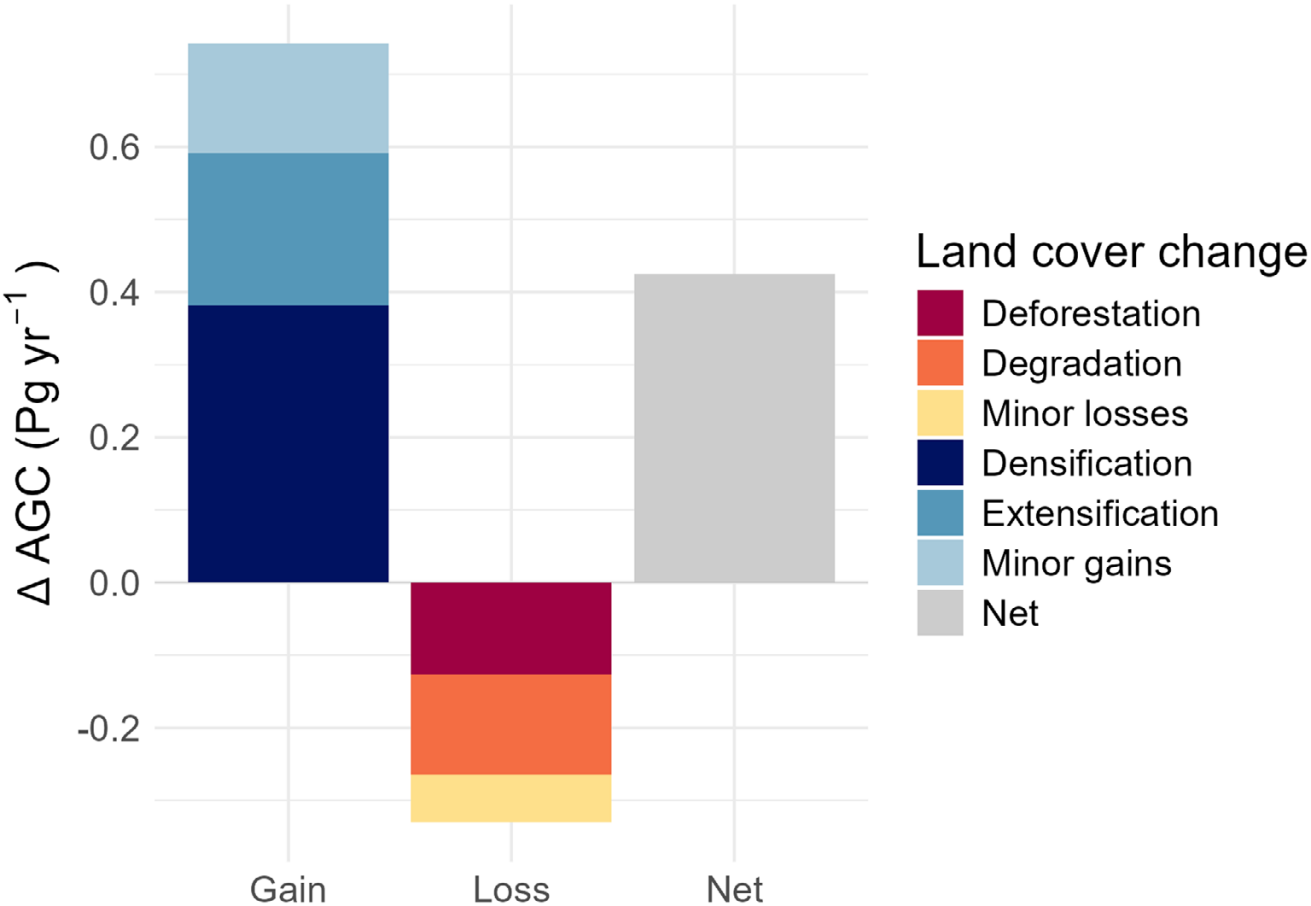
Contribution of different land‐cover change processes to the AGC change (2015–2024) across the full extent of dry forests and savannas. For the definitions of each land‐cover change process, see Section 2.7. Minor gains and losses are defined as changes of < 20% of the starting AGCD.

Gains were more common in untransformed landscapes. In contrast, losses were more common in transformed landscape units. Extensification was more widespread in untransformed land (18% of non‐wooded land) compared with transformed areas (13%) and densification was also proportionally more extensive in these areas (Table 2). The loss processes also differ greatly between transformed and untransformed land, with deforestation three times more common in transformed woody land than in untransformed areas. Degradation was also proportionally more extensive in transformed land, but the difference was smaller.

#### 3.5.2 Spatial Patterns and Combinations of Land‐Cover Change Processes

The spatial patterns of these different land‐cover change processes reveal very varied dynamics across the tropics (Figure 6). The hotspots of deforestation include the eastern parts of the humid and dry Chaco, where the agricultural frontier is moving west on both sides of the Paraguay‐Argentina border. Nigeria and Benin are also hotspots for deforestation, particularly across the southern parts of our study domain in these countries. We also find pockets of deforestation across the rest of the Chaco, the eastern Cerrado and south‐eastern Africa.

**FIGURE 6:**
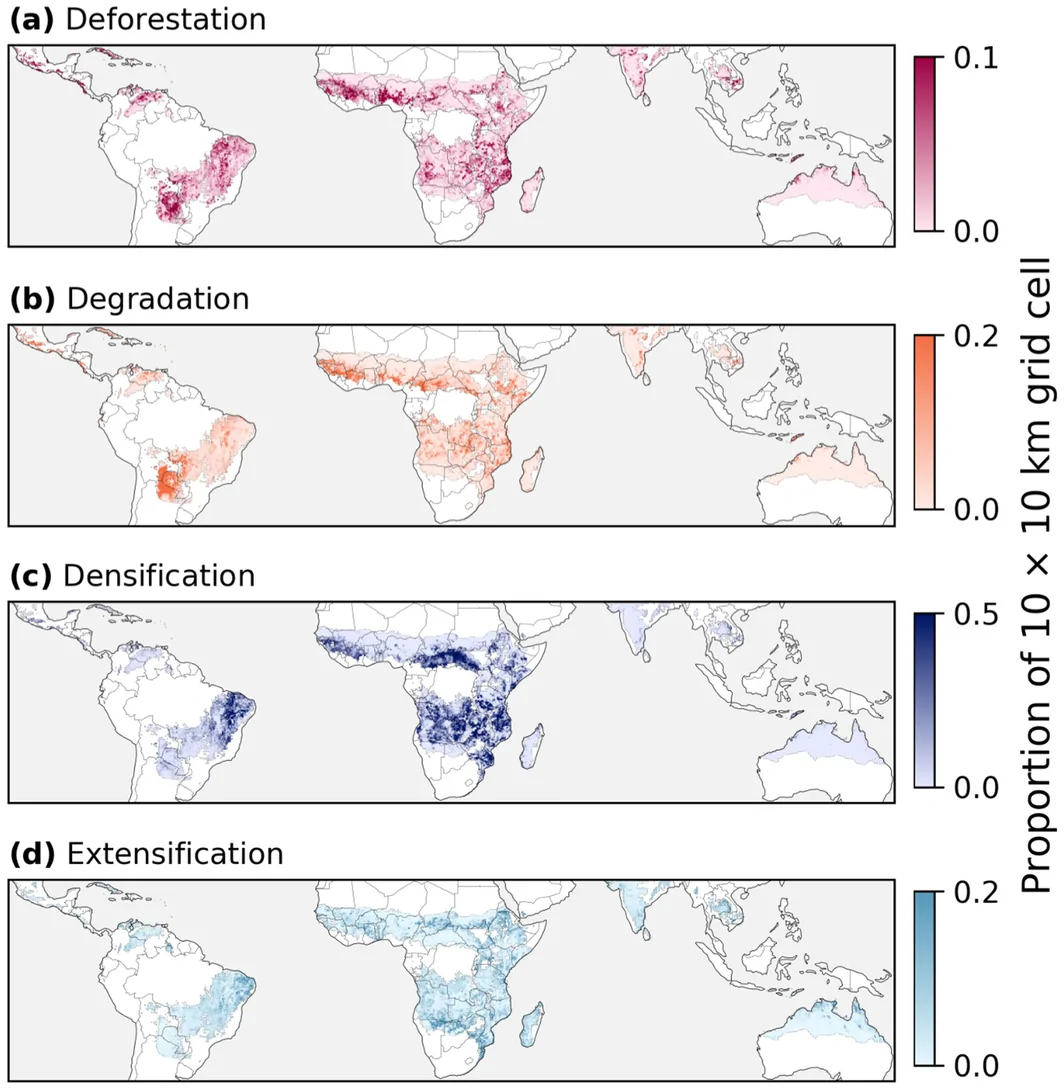
Pan‐tropical maps of land‐cover change processes at 10 km resolution: (a) deforestation in red, (b) degradation in orange, (c) densification in dark blue, and (d) extensification in light blue. Values are the proportion of each 10 km × 10 km grid cell undergoing the respective process over the study period (2015–2024). For visualisation, each panel is scaled so that the 95th percentile of that process' values maps to the maximum display intensity of its hue; note the different values of the maxima. Areas in white are outside the study domain. Map lines delineate study areas and do not necessarily depict accepted national boundaries.

Degradation is widespread across much of the western Chaco ahead of the agricultural deforestation frontier (Figure 7b), as well as across the more populated areas in west and southern Africa. Densification is prevalent across much of the dry tropics, but particularly in mid to high‐biomass wooded areas, including the Brazilian dry forests and the Caatinga, the northern Congo Basin forest‐savanna region, and the more remote areas of the Miombo.

**FIGURE 7:**
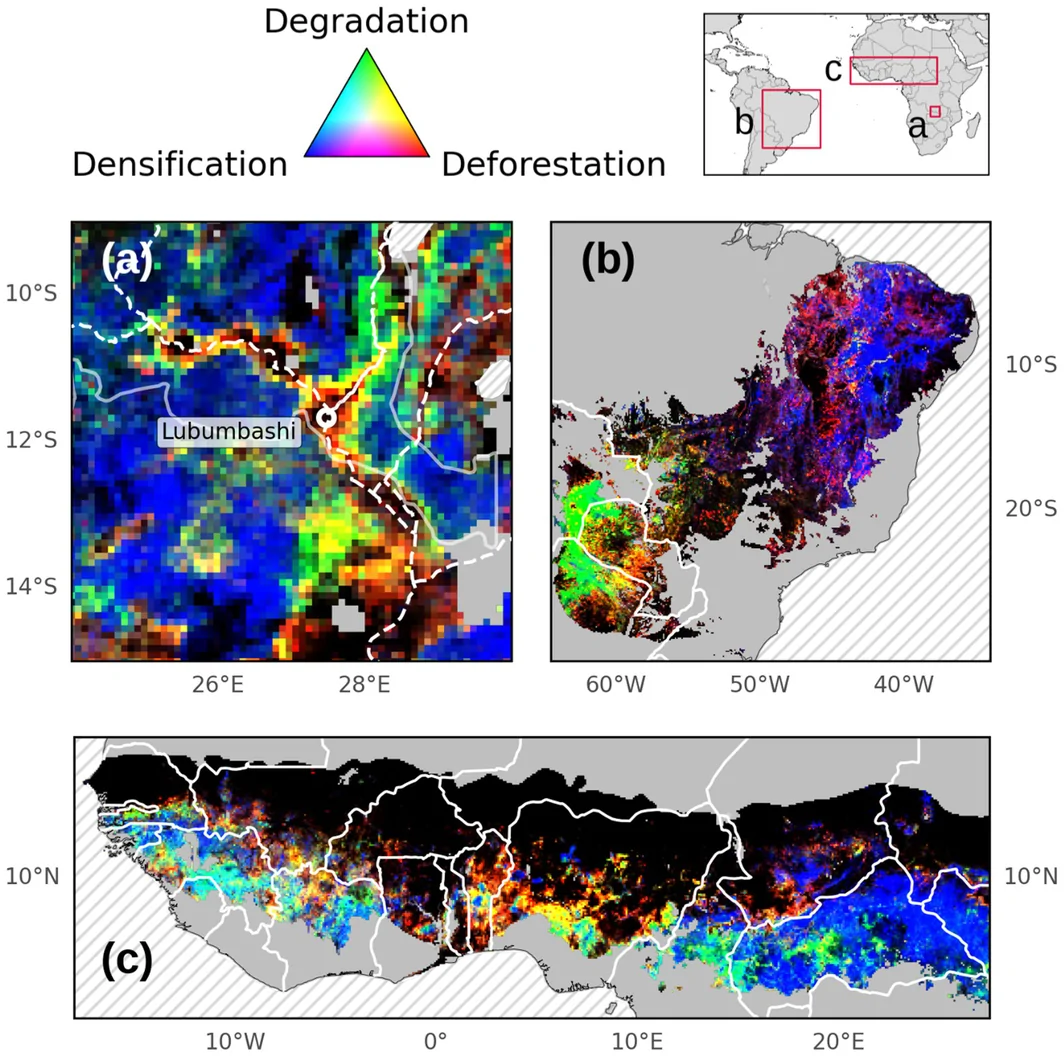
RGB composites of three wooded land‐cover change processes for (a) Lubumbashi, DRC; (b) the Dry Diagonal of South America; and (c) West Africa. As with Figure 6, each 10 × 10 km grid cell is coloured according to the extent of the land‐cover change process over the study period. Red encodes deforestation, green encodes degradation, and blue encodes densification. Intermediate colours indicate combinations of processes (e.g., yellow/orange = deforestation + degradation; cyan = degradation + densification; magenta = deforestation + densification). Black pixels indicate little or no occurrence of any process, while white indicates high, approximately equal values across all three. Channels are scaled per panel with maxima set to the 95th percentile of that channel's values within the panel extent for each region. White lines denote national borders, hatched areas denote oceans/lakes, dashed lines in panel (a) denote major roads. Maximum channel values in proportion of each 10 km × 10 km grid cell for (a) Def.: 0.10, Deg.: 0.20, Dens.: 0.65; (b) Def.: 0.10, Deg.: 0.20, Dens.: 0.40; and (c) Def.: 0.10, Deg.: 0.35, Dens.: 0.45. Map lines delineate study areas and do not necessarily depict accepted national boundaries.

The three woody‐land processes (deforestation, degradation, densification) often co‐occur, but in highly variable proportions (Figure 7). In south‐east Africa, there are clear waves of degradation followed by deforestation that radiate out of urban areas and along transport routes, particularly visible around Lubumbashi in the DRC (Figure 7a), Lichinga in northern Mozambique, and the road between Lusaka in Zambia and Lilongwe in Malawi. In West Africa (Figure 7c), there are both large areas where deforestation and degradation co‐occur (yellow and orange, centred on Nigeria and Benin) and areas where degradation and densification occur in close proximity (cyan, southern Guinea and northern Sierra Leone). The dry diagonal of South America (region of the continent spanning the Caatinga in northwest Brazil to the Chaco in Argentina) exhibits the full combination of these processes (Figure 7b), with degradation and deforestation in the south‐west (Chaco) giving way to increasing amounts of densification in the north‐east (Caatinga), while the Cerrado has a low but roughly equal level of all three processes underway.

Extensification (the shift from non‐wooded to wooded systems) is very widespread across several non‐wooded systems, with a large hotspot across the eastern half of the Sahel and an arc of extensification across the southernmost extent of the dry forest and savannas in Africa. Lesser amounts of extensification also occur in eastern parts of the Caatinga and in patches of northern Australia.

## 4 Discussion

We present an error‐bounded and validated pan‐tropical biomass‐backscatter relationship for dry forests and savannas calibrated and validated using a ground dataset of plots from dry forests and savannas. The resulting biomass maps provide a robust biomass and biomass change estimate for dry forest and savanna landscapes that can be used to address our research questions, as well as providing a publicly available data product.

This study provides methodological and conceptual advances. First, it provides plausible estimates of biomass change at a spatial and temporal resolution not previously achieved in dry tropical systems with the leading global product, CCI‐Biomass, flagging most of its pixel‐year estimates in our study area as improbable (only 4% of pixels in our domain have valid year‐to‐year change data). By contrast, this is the first product to map change across this extent with useful accuracy, and although the global carbon stock totals are broadly similar to CCI‐Biomass, the regional distribution differs substantially.

The underlying biomass‐backscatter model is calibrated and validated using a dry‐tropics‐specific field dataset of large, high‐quality research plots, addressing the calibration and validation gap that has previously limited the assessment of accuracy of global products. Our uncertainty assessment accounts for errors in these calibration and validation data, drawing on methods from both tree biometry and remote sensing. Secondly, the use of the multi‐temporal ALOS‐2 PALSAR‐2 ScanSAR acquisitions is an advance over the approach of McNicol et al. (2018, 2023). The temporal frequency of ScanSAR acquisitions means the resulting AGCD time series achieves greater temporal consistency than products built from the ALOS annual mosaics, probably due to reduced noise from soil moisture and individual acquisition artifacts. The 1 ha (100 m × 100 m) resolution is consistent with other leading biomass products and is a substantial improvement over the ~25 km resolution of passive microwave (L‐VOD) products. Finally, and with direct relevance to policy application, the change detection approach decomposes net AGC change into constituent processes (deforestation, degradation, densification and extensification). These categories are important to land managers and reporting under policy mechanisms such as REDD+ and the UNFCCC, which require the attribution of carbon loss to specific land‐use and land‐cover change processes. We now highlight the policy implications of these new data for restoration, conservation, land management and carbon accounting.

### 4.1 Importance of Tropical Dry Forests and Savannas in the Global Carbon Budget

Overall, dry forests and savannas play a substantial role in the global carbon cycle. They cover ~15% of the global ice‐free land area and our results show they make up a significant portion of global carbon stocks and the land carbon sink. The pan‐dry‐tropical AGC stock of 25.3 ± 2.0 PgC accounts for ~13.0% of global AGC stocks (Saatchi et al. 2011), and the net AGC increase of 0.43 ± 0.07 PgC yr^−1^ is 20.0% of the global net land flux (2.1 PgC yr^−1^; Friedlingstein et al. 2025), and 16.9% of the tropical ‘forest’ carbon sink (2.5 PgC yr^−1^; Pan et al. 2024). There is a relative absence of these systems on the global research agenda when compared with the humid tropics (Metcalfe et al. 2025; Schröder et al. 2021), but these results show that tropical dry forests and savannas warrant greater attention for their contribution to the global land sink, especially as these ecosystems face growing human and climatic pressures that could contribute to greater land‐use emissions and significantly alter their capacity to sequester carbon.

Our estimate for AGC stocks (25.3 ± 2.0 PgC) is higher than space‐borne LiDAR estimates (~15.6 PgC; L4B Biomass, with missing data filled with continental average biomass, converted to carbon; Dubayah et al. 2022), but is more similar to the radar‐based CCI‐Biomass product (version 6, ~23.2 PgC). Our estimate for the sink in the untransformed extent (0.36 PgC yr^−1^) is also similar to upscaled plot‐based estimates for the intact dry forests and savannas (0.22–0.43 PgC yr^−1^; Godlee et al. in review). This suggests there is now agreement between plot‐based methods and radar EO for estimates of the net sink. However, the EO data are able to map changes in the transformed landscapes, which are mostly unsuitable for long‐term plot measurements.

### 4.2 The Importance of Each Land‐Cover Change Process for Carbon Dynamics

It has been suggested that land‐use emissions have been underestimated because, beyond clear‐cut deforestation, they often exclude smaller‐scale management processes including wood‐fuel harvesting, shifting cultivation and other types of small‐scale agriculture and wood harvesting (Arneth et al. 2017; Prestele et al. 2017). This is particularly likely to be the case in the dry forests and savannas where shifting cultivation and wood harvesting are key livelihood activities driving land‐use emissions (McNicol et al. 2018; Ryan et al. 2016), particularly in Africa. The dynamics of these processes include frequent transitions in land cover and extensive, low‐intensity removal of biomass and are not fully captured in many global estimates of emissions. Consequently, there is a need to reevaluate land‐use emissions estimates by integrating improved carbon dynamics data that reflect the subtler impact of human activities on dry tropical landscapes.

Our results highlight that area‐density changes have a larger contribution to carbon changes than abrupt land‐cover changes, and confirm the importance of degradation as the major loss process in dry forests and savannas, with a degradation:deforestation ratio of 1.09 (0.14:0.13 PgC yr^−1^). These lower‐intensity, incremental carbon losses dominate removals across all of the largest dry forest and savanna systems in Africa and South America. Efforts to reduce emissions from deforestation and degradation (REDD+) have historically focussed more attention on preventing deforestation, particularly in intact forestland, in part due to deforestation being easier to quantify and monitor. However, this focus of resources on deforestation is missing significant potential land emissions reductions from avoiding degradation. Attention must move away from tropical deforestation, which, although symbolic, is probably not the main source of emissions (Qin et al. 2021). REDD+ interventions need robust data on the causes of woody biomass loss (Ryan et al. 2014). The spatially explicit information of biomass loss, particularly degradation, provided at administrative unit levels (in Section S9 and online at https://seco‐project‐2026.projects.earthengine.app/view/dry‐tropics‐biomass), will make it possible to analyse the drivers of biomass loss and develop appropriate policy responses (Bailis et al. 2015; Cotroneo et al. 2021; Lopes dos Santos et al. 2021; McNicol et al. 2018; Ryan et al. 2012; Schulz et al. 2018). Such policy responses need to carefully balance carbon‐based objectives and carbon‐focused definitions of ‘degradation’ with other values, including the trade‐off between biodiversity and carbon storage in many savanna and dry forest ecosystems (Parr et al. 2024; Ryan and Staver 2025).

Extensification—i.e., biomass gains in non‐wooded areas—is occurring across all continents, with 18.0% of non‐wooded untransformed land becoming wooded across the study period (2015–2024). This confirms fears of a pervasive shift away from open ecosystems (Stevens et al. 2017). These open systems represent a critical land cover for biodiversity and water. In untransformed dry forests and savannas, these non‐woody lands are generally grass‐dominated, disturbance‐driven biomes whose systems are shaped by fire and herbivory (Venter et al. 2018); alterations to these processes, as well as climatic influences like altered precipitation and CO_2_ concentrations, are likely to significantly affect their carbon dynamics (García Criado et al. 2020). The widespread gains we observe in these open systems may be interpreted as a growing part of the land sink of dry forests and savannas and represent a significant alteration to a biome with large impacts on ecology and livelihoods (Parr et al. 2024; Stevens et al. 2017).

### 4.3 Regional Variability in Carbon Dynamics and Land‐Cover Change Processes

The combination of varied ecological responses to global change, mediated by the functional diversity of a given region (Wells et al. 2025) and varied social and economic drivers of land use and land‐cover change, gives rise to large differences in the patterns and magnitudes of biomass change across continents and their major ecoregions. Because these drivers are highly local, regional land‐cover change patterns differ markedly, even when net carbon balances are similar. Changes occur at multiple scales: some processes operate broadly across regions, while others manifest as small, mixed‐process patches within landscapes. The spatial patterns of these land‐cover changes can provide information on the underlying drivers. Degradation remains comparatively understudied relative to deforestation (Feurer et al. 2025) and the drivers of encroachment (both extensification and densification) are not well understood (Ding and Eldridge 2024; Venter et al. 2018). Identifying drivers at multiple scales is essential for attributing emissions, designing targeted interventions and monitoring their impacts (Kissinger et al. 2012). Despite widespread deforestation and degradation across tropical dry forests and savannas, most major ecoregions, including the Caatinga, Cerrado and Miombo, continue to gain biomass, with the Chaco a notable exception. We now highlight some of the key regional patterns and likely drivers.

The Chaco in South America is well known as a hotspot for agricultural expansion and intensification, with dry forests being cleared for global commodity production, primarily cattle ranching and soybean cultivation (Baumann et al. 2017; Buchadas et al. 2022). We see this in our results, with the Chaco losing −2.8% of its carbon stocks annually, with degradation and deforestation more extensive than in other major ecoregions (Figure 7). We see widespread degradation in the western (mostly dry) Chaco, beyond the deforestation of the agricultural frontier in the eastern (humid) Chaco. While deforestation is occurring at the agricultural frontier in sites where intensive cattle ranching is taking place, the pattern of degradation corresponds with the distribution of more extensive, low‐stocking‐density smallholder and semi‐subsistence woodland cattle production systems (Fernández et al. 2020). Degradation may also be a response to drought and reduced water availability (Bracalenti et al. 2024; De Marzo et al. 2021).

Despite transformation and human use across the Caatinga, we find that it remains an AGC sink and is experiencing rapid carbon gains at 4.4% a year. This supports results from Mendes et al. (2025), who found that the Caatinga's high carbon‐use efficiency makes it a very effective carbon sink. However, the region is experiencing widespread human and climatic pressures (Araujo et al. 2023), which may compromise this sink.

Africa dominates dry forest and savanna carbon stocks and fluxes. Existing remotely sensed products have not been well parametrised for African ecosystems and ground data are sparse, so accurate monitoring of carbon stocks and fluxes across the continent has been limited (Ernst et al. 2024). There is also increasing attention on and controversy over using African dry tropical ecosystems for carbon storage and offsets (Kateka 2025). The majority of the global dry forests and savannas are in Africa (60% of the pan‐tropical extent) and these dry forests and savannas store proportionally more carbon and are accumulating carbon at a faster rate than other regions. Africa stores 66.0% of the pan‐tropical dry forests and savanna stock, and 72.0% of untransformed carbon stocks. Of the 0.74 PgC yr^−1^ of gross woody gains across the tropics, 69.0% are occurring in Africa. These gains are both extensive and intensive, with gains occurring across a greater extent of the continent than other continents, as well as occurring at a greater per hectare rate, including in the transformed landscapes. Concomitantly, rates of carbon loss from deforestation and degradation are lower across Africa, with 50.0% of pan‐tropical deforestation and degradation occurring in Africa. The predominant drivers of losses across Africa are small‐scale (wood harvesting, clearance for small‐scale agriculture, or shifting cultivation; Curtis et al. 2018; Ryan et al. 2014), but, even in the hotspots of losses, they are generally lower and part of mixed‐process mosaics than in other parts of the tropics.

In some of the regions in Africa, we see relatively large net losses and a patchwork of deforestation and degradation, most prominently in Nigeria and Benin (Figure 7), where net AGC losses exceed 2% a year. These losses across Nigeria and Benin are also seen in L‐VOD data analyses (Brandt et al. 2018; Wang et al. 2024; Zhao et al. 2024). The local socio‐economic context leads to land cover dynamics typical of small‐scale agricultural expansion (Brandt et al. 2018; Buchadas et al. 2022; Lourenço et al. 2025; Wang et al. 2024; Wingate et al. 2024), with both deforestation and degradation processes happening concurrently (Figure 7c, yellow and orange). The nature of these drivers means they are likely to be occurring in close proximity (Lourenço et al. 2025), as we see in our results.

The Miombo woodlands in Southern Africa are the largest savanna complex in the world (Mistry and Berardi 2014). Our estimates of aboveground carbon stock in the Miombo ecoregion are consistent with findings from a regional study with high‐resolution data which found small net gains in the period between ~2007 to ~2015 (McNicol et al. 2023), and a coarse‐resolution continental study covering the period 2003–2021 (Wang et al. 2024). We model small‐scale patterns of degradation in hotspots surrounding some large population centers and along transport routes across Southern Africa, where a radial wave of degradation is followed by deforestation (Figure 7a). This pattern is also seen in a regional study (McNicol et al. 2018) that suggests these patterns of degradation are often the result of timber resource extraction for local or international markets, as well as charcoal production to support the energy demands of nearby towns and cities rather than subsistence (Ahrends et al. 2010; McNicol et al. 2018; Mitchard et al. 2013; Ryan et al. 2012). Despite these carbon removals, the ecoregion is experiencing net AGC gains of over 2% a year. Gains here, and across the continent, could, in part, be the result of widespread cropland abandonment in many parts of the continent (Lou et al. 2025).

Similarly, we observe strong AGC increases in the Central African Republic of around 2% a year, gains which are also observed in similar studies using coarse‐resolution L‐VOD data (Brandt et al. 2018; Wang et al. 2024; Zhao et al. 2024). This trend may be the result of a low population density and subsequent low local pressures on wood resources, as well as long‐term political instability resulting in emigration from large areas of the country and deterring large‐scale deforestation activities (Wang et al. 2024).

Our finding of widespread encroachment is seen in other studies (Axelsson and Hanan 2018; García Criado et al. 2020; Stevens et al. 2017; Venter et al. 2018), and the ‘Arc of Extensification’ in Southern Africa has been attributed to increasing atmospheric CO_2_, changes in land management, fire and climate (Stevens et al. 2017; Venter et al. 2018). There is less agreement about the scale of extensification happening in the other hotspot we identified across the eastern Sahel, with another remote sensing study showing a mixture of losses and gains (Venter et al. 2018) and a net loss in carbon estimated from upscaled estimates from plot data (Godlee et al. in review). There is little literature on the vegetation dynamics of the dry forests and savannas of the eastern Sahel, as well as a lack of ground data. Extensification is a response to changing environmental conditions or disturbances, and many of these drivers occur at a macro scale. However, highlighting spatial patterns of extensification may help in facilitating improved management of these systems (Ding and Eldridge 2024) that do not degrade the open‐ecosystem functions and services that support biodiversity and millions of rural livelihoods. Carbon‐focused land management policies that incentivise carbon accumulation without distinguishing between forest recovery and the conversion of fire‐adapted grasslands promote ecologically damaging land‐cover transitions. Effective policy must therefore move beyond carbon to encompass the full suite of ecosystem values at stake in these biomes.

These results also highlight how dynamic these biomes are. Part of what we term deforestation, degradation, densification and extensification here likely sits within the natural variability of these ecosystems. In savannas in particular, rapid shifts between woody and open states have likely occurred throughout their evolutionary history (Gillson 2004), driven by fire, herbivory and climatic variability (Daskin et al. 2016; Dublin et al. 1990). Even at the shorter timescales considered here, this dynamism may persist. For example, elephant browsing or herbivory can open up closed‐canopy woodland in a matter of years, which may then return to woody states within a decade or two (Beuchner and Dawkins 1961). In areas where these natural processes still dominate vegetation change, our land‐cover change processes as defined here can thus sometimes be a shift in tree‐grass ratios rather than a change or loss of the whole ecosystem. This raises an issue with the terminology itself, as ‘deforestation’ and ‘degradation’ carry negative implications, while ‘densification’ and ‘extensification’ are comparatively neutral, despite being symmetric descriptions of biomass change. Finding the right vocabulary that avoids embedding value judgements into these processes is a challenge, and we recognise that our terminology is not exempt from this critique. What we term ‘degradation’ is not inherently damaging in much of this domain, and ‘extensification’ can be detrimental to ecosystem function in open systems. We do not propose an alternative here, but believe the research and policy community would benefit from developing terminology that better reflects the distinction between the natural dynamism of these ecosystems and the human‐imposed land‐cover changes, rather than treating all biomass loss as undesirable and all biomass gain as positive.

Southeast Asia has some of the fastest gains and greatest losses. With almost all of the subcontinent dominated by ‘transformed’ landscapes (96%), much of these dynamics are driven by plantation rotation for global commodities (Buchadas et al. 2022) like rubber, which can be fast‐growing and store substantial carbon stocks when mature (Tiko et al. 2025; Wigneron et al. 2024). Southeast Asia is exceptional in the scale of the gains and losses in transformed land (Section S9); in the other continents, most gains by area and carbon mass are in untransformed, already wooded landscapes. Non‐transformed wooded areas should remain a key component of conservation and REDD+ efforts preventing biomass loss in existing wooded areas.

Tropical dry forests and savannas have undergone significant transformation, predominantly being converted into agricultural landscapes or mosaic agricultural landscapes. Despite the extensive deforestation and degradation that accompany transformation, these agricultural landscapes still hold significant stocks of C and continue to accumulate biomass (Table 2). Across the tropics, much of these transformed landscapes are complex mosaics of land uses which include patches of remaining trees and regrowing or fallow land, developed through practices such as shifting cultivation and selective clearance (van Vliet et al. 2012). In addition, some of the highest rates of biomass accumulation are observed within transformed landscapes with a rotation of plantation crops (Section S4). This systematic planting and harvesting can lead to highly dynamic landscapes with mosaics of substantial biomass accumulation and clearances. These observed trends highlight the need for land management and interventions that recognise the potential for biomass retention and accumulation in agricultural landscapes. The inevitability of continued land transformation across parts of the dry forests and savannas means policies that support sustainable agricultural practices can have a meaningful impact on carbon storage.

Our results indicate that dry forests and savannas currently function as a net AGC sink. Over the coming decades, the balance of losses and gains will be shaped by interacting biophysical and human drivers: ongoing land transformation, alongside changing farming systems and technologies, and changes to energy demand and sources (Ernst et al. 2024; Masolele et al. 2024; McNicol et al. 2018; Ryan et al. 2012); changing temperatures and precipitation that affect tree growth, mortality and recovery; shifts in disturbance regimes (fire and herbivory; Andela et al. 2017; Sankaran et al. 2008; Stevens et al. 2017); and CO_2_ fertilisation (Archer et al. 2017). If protection of untransformed landscapes is strengthened and woodfuel and small‐scale agriculture transition toward alternative practices, the sink could be maintained. Conversely, losses from continued land transformation and degradation could increasingly offset gains, risking regional or global sink weakening or reversal.

Several limitations should be considered when interpreting our results. Although we have assembled a large calibration and validation dataset for these biomes, it is still relatively sparse in some regions, particularly Asia (Section S5.1). In addition, the biomass‐backscatter model only explains 56% of the deviance, so there may be larger uncertainties in the regions where plot data are sparse and where environmental conditions (presumed to be the source of the unexplained deviance) are different from the calibration data set. For roughly 20% of the domain, mostly in Australia, ScanSAR coverage was insufficient to carry out our analysis and we relied on the ALOS PALSAR‐2 annual mosaic product to gap‐fill, introducing additional, unquantified uncertainty, as this product only uses one SAR observation a year. While our stock estimates were validated against held‐back plot data, we lack an equivalent independent validation of Δ AGCD, a current limitation of all biomass change mapping. The short observational record of ScanSAR (< 10 years for PALSAR‐2) limits our ability to detect longer‐term trends or any non‐linearity of these trends, and the change derived from the slope of a linear regression does not capture the shorter scale year‐to‐year variability (although it is presented as an annualised figure, this is the mean of the change over the time series and is not intended to capture year‐to‐year change). Finally, our land transformation definition does not capture transformations such as grazing by domesticated animals or invasive species dominance, which are difficult to map at a global scale, so some land we term ‘untransformed’ may still be under significant human or ecological pressure.

## 5 Conclusion

Tropical dry forests and savannas are a substantial and dynamic component of the global carbon budget. Using a dry‐tropics‐specific SAR‐biomass model, we map stocks, fluxes and land‐cover change processes across the tropics. Our results show that these ecosystems hold a large share of global aboveground woody carbon and act as a significant land sink. This domain‐specific approach provides reliable estimates for monitoring carbon changes in regions where data have been sparse or inaccurate, providing an important resource for conservation, restoration and carbon accounting.

Spatially, carbon change occurs as both large‐scale regional changes and fine‐grained mosaics governed by local ecological and socioeconomic drivers. Carbon losses are driven equally by degradation and deforestation, highlighting the pressures on woody resources from woodfuel, timber and other tree harvesting. Gains arise both from the densification of existing wooded, mainly untransformed, areas and from the extensification of non‐wooded systems into wooded lands. Densification occurs mostly in relatively undisturbed forests and savannas and contributes strongly to the sink. Extensification is very widespread, signalling a pervasive shift away from open ecosystems, with implications for biodiversity and livelihoods. Collectively, these findings suggest that land management and policy will need to evolve to meet the challenges of global change in these biomes and explicitly address trade‐offs between biodiversity, livelihoods and climate change mitigation.

## Author Contributions

**Sam B. Harrison:** conceptualization, data curation, formal analysis, investigation, methodology, validation, visualization, writing – review and editing, writing – original draft. **John L. Godlee:** writing – review and editing, methodology, investigation, formal analysis. **David T. Milodowski:** formal analysis, methodology, writing – review and editing. **Reza Asiyabi:** writing – review and editing. **Penelope J. Mograbi:** writing – review and editing, data curation, investigation, project administration. **Zhofre Aguirre:** investigation, writing – review and editing. **Jarcilene S. Almeida:** investigation, writing – review and editing. **George K. D. Ametsitsi:** investigation, writing – review and editing. **Alejandro Araujo‐Murakami:** investigation, writing – review and editing. **Sally Archibald:** investigation, writing – review and editing. **Luzmila Arroyo Padilla:** investigation, writing – review and editing. **Lorena Benitez:** investigation, data curation, writing – review and editing. **Samuel J. Bowers:** writing – review and editing, investigation. **Thomas K. Brade:** investigation, writing – review and editing. **Domingos Cardoso:** investigation, writing – review and editing. **João M. B. Carreiras:** writing – review and editing. **Antonio Alberto Jorge Farias Castro:** investigation, writing – review and editing. **Duncan Chalo:** investigation, writing – review and editing. **Halidou Compaore:** investigation, writing – review and editing. **Isabela S. C. da Costa:** investigation, writing – review and editing. **Ítalo Antônio Cotta Coutinho:** investigation, writing – review and editing. **Eder C. das Neves:** investigation, writing – review and editing. **Vera De Cauwer:** investigation, writing – review and editing. **Tony César de Sousa Oliveira:** investigation, writing – review and editing. **Hermane Diesse:** investigation, writing – review and editing. **Tomas F. Domingues:** investigation, writing – review and editing. **Fernando Elias:** investigation, writing – review and editing. **Camila Laís Farrapo:** investigation, writing – review and editing. **G. Feig:** investigation, writing – review and editing. **Ted R. Feldpausch:** investigation, writing – review and editing. **Moabe F. Fernandes:** investigation, writing – review and editing. **David R. Galbraith:** investigation, writing – review and editing. **Nestor I. Gasparri:** investigation, writing – review and editing. **Emanuel Gloor:** investigation, writing – review and editing. **Francisco Maiato:** investigation, writing – review and editing. **Tatenda Gotore:** investigation, writing – review and editing. **Karollyne S. Guimarães:** writing – review and editing, data curation. **Gloria Gutierrez‐Sibauty:** investigation, writing – review and editing. **Rhett D. Harrison:** investigation, writing – review and editing. **Thomas P. Higginbottom:** writing – review and editing. **Francoise Yoko Ishida:** investigation, writing – review and editing. **Hamza Issifu:** investigation, writing – review and editing. **John Ngugi Kigomo:** investigation, writing – review and editing. **Timothy J. Killeen:** investigation, writing – review and editing. **Aurora Levesley:** data curation, project administration, writing – review and editing. **Simon L. Lewis:** data curation, investigation, writing – review and editing. **José Romualdo S. Lima:** investigation, writing – review and editing. **Dante Loto:** investigation, writing – review and editing. **Elisvan Mariano Sousa:** investigation, writing – review and editing. **Beatriz S. Marimon:** investigation, writing – review and editing. **Ben Hur Marimon Junior:** investigation, writing – review and editing. **Mário Marcos do Espírito Santo:** investigation, writing – review and editing. **Antonia M. L. Marques:** investigation, writing – review and editing. **Matheus Santos Martins:** investigation, writing – review and editing. **Collins W. Masinde:** investigation, writing – review and editing. **Musingo Tito E. Mbuvi:** investigation, writing – review and editing. **Iain M. McNicol:** investigation, writing – review and editing. **Karina Melgaço:** data curation, writing – review and editing. **B. Mogonong:** investigation, writing – review and editing. **Peter W. Moonlight:** investigation, writing – review and editing. **Paulo S. Morandi:** investigation, writing – review and editing. **Leonor Patricia Cerdeira Morellato:** investigation, writing – review and editing. **Magna Soelma Beserra de Moura:** investigation, writing – review and editing. **Anderson Muchawona:** investigation, writing – review and editing. **Jonathan Ilunga Muledi:** investigation, writing – review and editing. **Leena Naftal:** investigation, writing – review and editing. **Jhonathan Oliveira Silva:** investigation, writing – review and editing. **Catherine L. Parr:** investigation, writing – review and editing. **Nayane Cristina C. S. Prestes:** data curation, investigation, writing – review and editing. **Luciano Paganucci de Queiroz:** investigation, writing – review and editing. **Tshililo Ramaswiela:** investigation, writing – review and editing. **Desirée M. Ramos:** investigation, writing – review and editing. **Simone Matias Reis:** investigation, writing – review and editing. **Natasha S. Ribeiro:** investigation, writing – review and editing. **Raphael R. Rocha:** investigation, writing – review and editing. **Priscyla Maria Silva Rodrigues:** investigation, writing – review and editing. **Gustavo Saiz:** investigation, writing – review and editing. **Rubens M. Santos:** investigation, writing – review and editing. **Mylor Ngoy Shutcha:** investigation, writing – review and editing. **Domingos Fortunato P. F. da Silva:** investigation, writing – review and editing. **Kathleen G. Smart:** investigation, writing – review and editing. **Bonaventure Sonké:** investigation, writing – review and editing. **Elmar Veenendaal:** investigation, writing – review and editing. **Chemuku Wekesa:** investigation, writing – review and editing. **Lucy H. Wells:** investigation, writing – review and editing. **Débora Zuanny:** investigation, writing – review and editing. **Timothy R. Baker:** conceptualization, data curation, funding acquisition, investigation, writing – review and editing. **Kyle G. Dexter:** conceptualization, funding acquisition, investigation, project administration, writing – review and editing. **Gabriele C. Hegerl:** conceptualization, funding acquisition, writing – review and editing. **R. Toby Pennington:** conceptualization, funding acquisition, investigation, project administration, writing – review and editing. **Oliver L. Phillips:** conceptualization, data curation, funding acquisition, investigation, project administration, writing – review and editing. **Stephen Sitch:** conceptualization, funding acquisition, writing – review and editing. **Ann Carla Staver:** conceptualization, writing – review and editing. **Mathew Williams:** conceptualization, funding acquisition, investigation, writing – review and editing. **Shaun Quegan:** conceptualization, funding acquisition, methodology, writing – review and editing, project administration, supervision. **Steven Hancock:** conceptualization, writing – review and editing, methodology, supervision. **Nicole Helene Augustin:** conceptualization, writing – review and editing, formal analysis, methodology, supervision. **Casey M. Ryan:** conceptualization, writing – review and editing, data curation, formal analysis, funding acquisition, investigation, methodology, project administration, supervision, visualization, validation, writing – original draft.

## Funding

This work was supported by the Natural Environment Research Council, NE/T01279X/1.

## Conflicts of Interest

The authors declare no conflicts of interest.

## Supporting information


**Figure S1:1** Number of ScanSAR images in each year 2015–2024. Map lines delineate study areas and do not necessarily depict accepted national boundaries.
**Figure S1:2** Day of the year of the minimum backscatter value for each year 2015–2.
**Table S1:1** ScanSAR masking parameters by region.
**Figure S2:1** The global extent of dry forests and savannas, classified into untransformed (dark blue) and human‐transformed (orange) landscapes. The definition of transformed is based on land‐cover information only, and identifies landscape units mostly comprised of anthropic land covers or based on land cover maps and a tree‐cover loss product. Map lines delineate study areas and do not necessarily depict accepted national boundaries.
**Figure S2:2** Largest Ecoregions in the dry forests and savannas. Map lines delineate study areas and do not necessarily depict accepted national boundaries.
**Figure S3:1** Distribution of plots (*n* = 237, black dots) used in the AGCD modelling across the dry forest and savanna domain (orange). Map lines delineate study areas and do not necessarily depict accepted national boundaries.
**Table S4:1** Contribution of plantation areas to pan‐tropical aboveground carbon dynamics in savannas and dry forests. Areas demarcated as plantations were devided into areas with positive Δ AGCD (gains) and negative Δ AGCD (losses) based on the slope of the linear regression of time vs. AGCD (2015–2024).
**Table S5:1** Model summary statistics for the Generalised Additive Model (GAM) fitted to aboveground woody carbon density (AGCD) using radar backscatter (HV) in the dry tropics, including estimated degrees of freedom (edf), F‐statistics, and model fit.
**Table S5:2** Biomass range‐specific validation statistics (Systematic deviation (Equation 2) and Root Mean Squared Deviation (RMSD; Equation 3)) following Duncanson et al. (2021).
**Figure S5:1** Validation of the AGCD‐backscatter model using holdout sites, grouped by continent. The black line indicates 1:1.
**Table S5:3** Continent‐specific validation statistics (Systematic deviation (Equation 2) and Root Mean Squared Deviation (RMSD; Equation 3)).
**Figure S5:2** Validation of the AGCD‐backscatter model using holdout sites, grouped by biome. The black line indicates 1:1.
**Figure S5:4** Biome‐specific validation statistics (Systematic deviation (Equation 2) and Root Mean Squared Deviation (RMSD; Equation 3)).
**Figure S5:3** Uncertainty in the biomass‐backscatter relationship across the range of radar data observed in the training/testing data. Uncertainty is estimated with an end‐to‐end Monte Carlo procedure that propagates three error sources: uncertainty in plot‐based AGCD (from biometry, allometry, wood density), uncertainty in the annual minimum backscatter (modelled as a normal distribution with σ=~12% of the mean, estimated from overlapping ScanSAR scenes), and statistical uncertainty in the GAM that links backscatter to AGCD.
**Figure S6:1** Validation of the AGCD‐backscatter model using holdout sites, and the validation of the CCI‐Biomass v6 data product using the same plots. The dashed line indicates 1:1; the blue line shows the linear fit of our model, and the red line shows the CCI‐Biomass linear fit. RMSD = Root Mean Squared Deviation. SD = Systematic Deviation.
**Table S6:1** Biomass range‐specific validation statistics (Systematic deviation (Equation 2) and Root Mean Squared Deviation (RMSD; Equation 3)) for the CCI‐Biomass v6 data product against the plot data used in our GAM model, following Duncanson et al. (2021).
**Figure S6:2** Spatial comparison of our estimates of mean AGCD with the CCI‐Biomass product (version 6) by Ecoregion (Dinerstein et al. 2017). Positive values (blue) indicate where CCI‐Biomass estimates higher mean ecoregion AGCD than this study.
**Figure S8:1** Map showing areas where biomass is predicted from ScanSAR data (blue) and where it is gap‐filled using the mosaic model (orange). Map lines delineate study areas and do not necessarily depict accepted national boundaries.
**Table S8:1** Summary statistics for the mosaic model used to gap‐fill. The Generalised Additive Model (GAM) is fitted to aboveground woody carbon density (AGCD) from ALOS‐2 PALSAR‐2 annual mosaic radar backscatter as a predictor variable in the dry tropics.
**Figure S8:2** The GAM mosaic model was validated in the same was as the main model. Validation of the AGCD‐mosaic backscatter model using holdout sites. Points show the field‐estimated AGCD and the mosaic model prediction. The dashed line indicates 1:1; the thick red line a GAM smooth and the blue line the linear fit. RMSD = Root Mean Squared Deviation. The model predictions are based on a leave‐one‐site‐out procedure.

## Data Availability

Data will be published in a public repository that issues datasets with DOIs upon acceptance of the paper. Data will be shared with reviewers upon request.
