# Supplementary material for: Carbon Accumulation in Tropical Dry Forests and Savannas Despite Widespread Land‐Cover Change, Revealed by Radar Remote Sensing
Source: Glob Chang Biol. 2026 Oct 19;32(10):e71141. doi: 10.1111/gcb.71141 (PMC13680975; doi:10.1111/gcb.71141)
Supplement: Supplementary file 1 [file GCB-32-e71141-s001.pdf]

Supplementary Materials

S 1 Radar data

S 1.1 ScanSAR observations

ALOS-2 PALSAR-2 ScanSAR acquisitions nominally provide repeat coverage over the tropics every ~42 days, but realised observation frequency varies substantially in space and time. The number of images per pixel-year ranged from none to several dozen in places where overlapping swaths and repeated passes were available (Figure S 1.1).

For each pixel-year, we extracted the annual minimum HV backscatter to minimise the influence of soil moisture on radar response in these seasonal systems. The day-of-year (DOY) of these minima follows expected regional seasonality, clustering according to local dry season climatology, but with year-to-year variability reflecting both interannual climate fluctuations and acquisition schedules (Figure S 1.2). This spatial and temporal heterogeneity in observation counts informed the continent-specific masking. Where observation frequency fell persistently below threshold levels (defined in table S 1.1), notably across Australia, we complemented the ScanSAR-based predictions with a secondary model based on annual ALOS PALSAR mosaics.

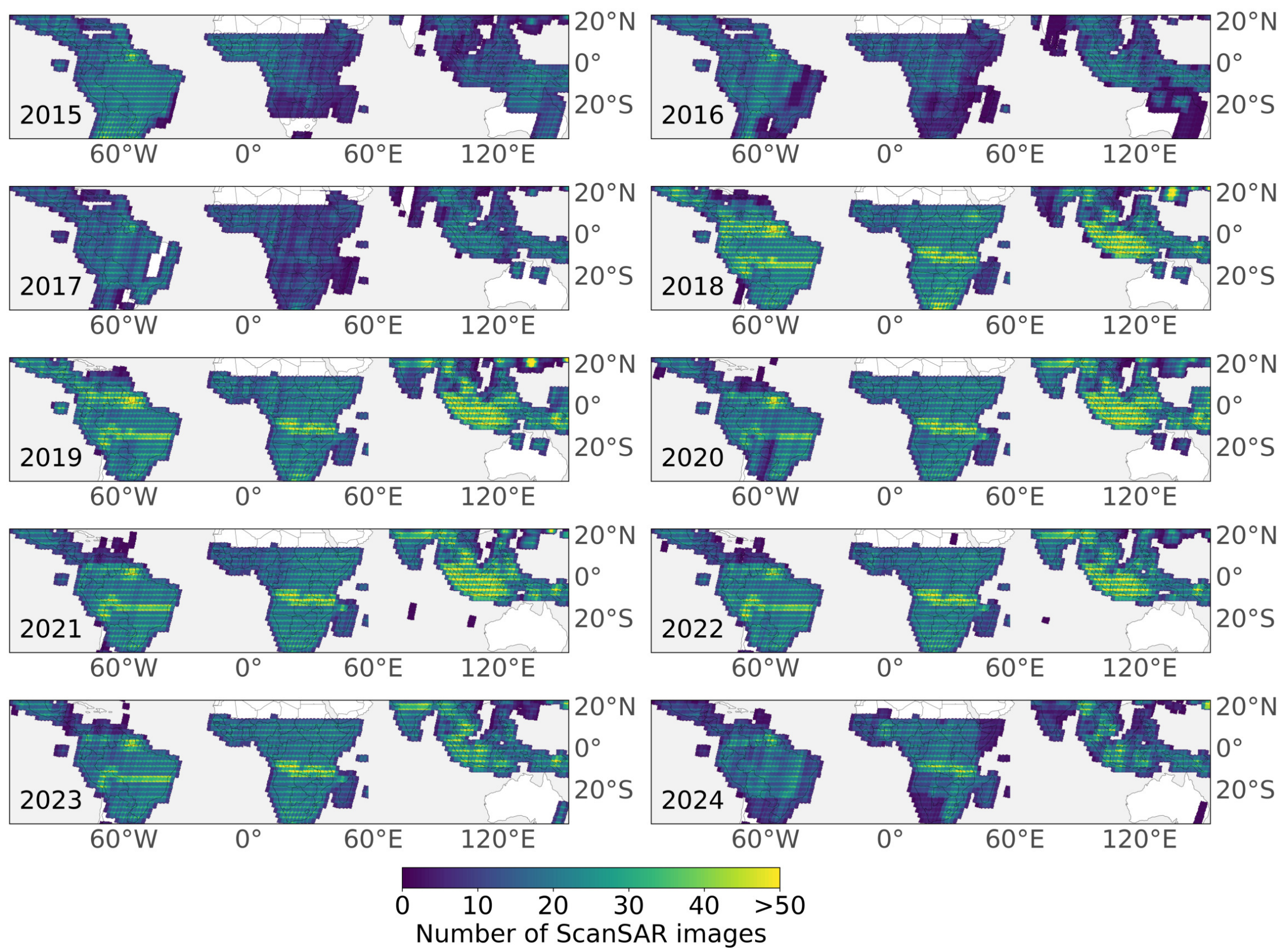

Figure S 1.1: Number of ScanSAR images in each year 2015–2024. Map lines delineate study areas and do not necessarily depict accepted national boundaries.

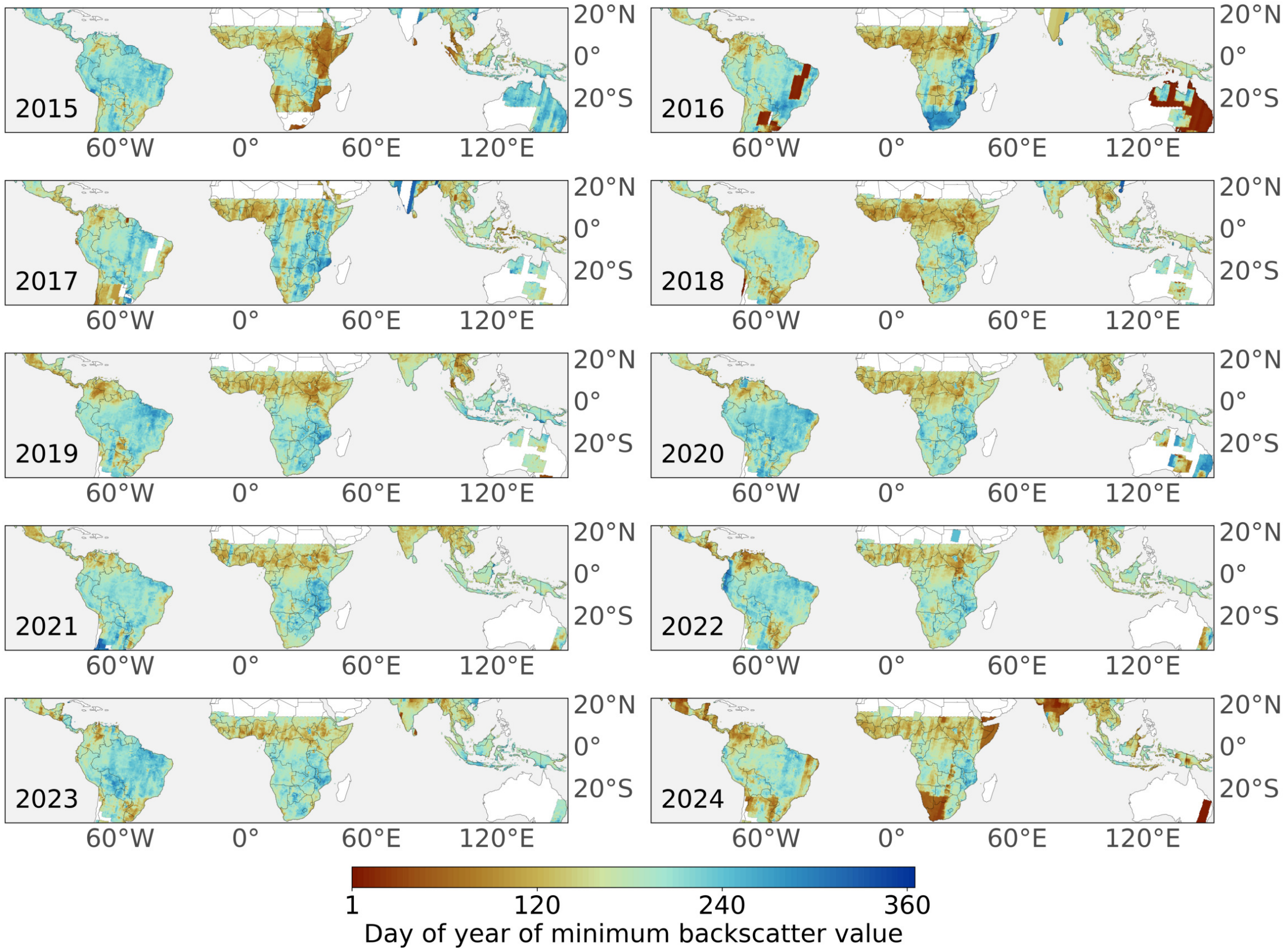

Figure S 1.2: Day of the year of the minimum backscatter value for each year 2015–2024. Map lines delineate study areas and do not necessarily depict accepted national boundaries.

S 1.2 ScanSAR quality masking

We used continent-specific masking as each continent had different ScanSAR data provision and quality issues. A pixel was masked for a given year based on the number of ScanSAR observations in the year and the number of months in the year with at least one image. Problematic years, identified as years with consistent outliers in the time series, were removed entirely. Pixel-years with high soil moisture were also masked. We used ERA5-Land Daily Aggregated volume of water in soil surface (0-7 cm) on the day of the annual minimum backscatter (ECMWF Climate Reanalysis, Muñoz-Sabater et al., 2021) to mask out pixel-years with high soil moisture (>0.3 m^3 m^{-3}), as soil moisture above this level suggests flooded or inundated land not suitable for AGCD estimation from SAR (Gou et al., 2022; McNicol et al., 2018). Due to the varying data quality across each continent, some areas have fewer years of data to draw from, with all regions having data from at least 2018-2021.

Table S 1.1: ScanSAR masking parameters by region

Region	Minimum no. images in year	Maximum soil moisture (fraction of water in soil volume)	Minimum no. of months with an image	Years	Minimum no. of unmasked pixels in time series
Central America	14	30	10	2018-2021	3
South America	9	35	9	2018-2024	3
Africa	6	38	6	2018-2024	3
India	6	30	6	2018-2021	4
Mainland SE Asia	5	45	5	2015–2023	3
Insular SE Asia	20	30	10	2015–2021	4

S 2 Domain

To delineate vegetated tropical lands we first excluded arid regions with very low woody AGCD by removing areas with <3 wet months (defined as <30 mm total monthly rainfall based on ERA5-Land; Muñoz-Sabater et al., 2021). Next, we excluded areas with Mediterranean and temperate climates which are likely to include other ecosystems (coolest quarter mean temperature <15 °C; Fick and Hijmans, 2017), and extra-tropical regions north of the Tropic of Cancer. Within these climate bounds, we identify vegetation that is likely dry forest or wooded savannas using land cover data and the Ecoregions dataset (Dinerstein et al., 2017). The Ecoregions dataset maps ecosystems of regional extent that share similar environmental conditions and species assemblages. By working at a landscape scale (10×10 km) we sought to overcome challenges of edge effects and multiple spatial scales of relevant land cover information. Landscape units were excluded if the sum of the tropical rainforest and non-vegetated land cover classes at 30 m resolution was in the majority. These land covers are based on the land covers from the Copernicus land cover product (evergreen closed canopy forest classes, wetlands, water and urban from CGLS-LC100; Buchhorn et al., 2020), and ecoregions (Mangrove forests, Coniferous Forests, and Moist Broadleaf Forests ecoregions except African coastal forests and Madagascan subhumid forests from Dinerstein et al., 2017). Some flooded land and newly constructed reservoirs were not picked up by the Copernicus land cover product for 2019. As such, some additional masks were applied to the maps. Using the MODIS Land Cover product (MCD12Q1) for 2024, if a pixel was classed as water in any of its classification schemes, it was masked out. This removed most water bodies missed by the Copernicus product. The Sudd wetlands are not well mapped by land cover products due to large interannual variation in flooding. Instead of a remote-sensing based product, we used the Terrestrial Ecoregions of the World data (Dinerstein et al., 2017) to remove the wider Sudd flooded grasslands ecoregion from the map. A surface water and urban land cover mask was applied again at higher resolution to the remaining land to ensure these land covers are not included in zonal statistics.

We split the landscape units into 'untransformed' and 'human-transformed' using the Copernicus land cover product (Figure S 2.1). We define landscape transformation using forest loss and land cover data, categorising a landscape unit as "transformed" if the majority of the landscape is classed as cropland, has a cover fraction of cropland over 30% (Buchhorn et al., 2020), or has experienced tree-canopy loss since 2000 according to Hansen et al., 2013. Transformed landscapes may therefore include large-scale commercial agriculture, smallholder agricultural systems, mosaics of agriculture among remnant forest or secondary forest, or cleared and abandoned land. Untransformed landscapes may still experience significant human pressures and clearance, but the land cover has not been changed to a predominantly anthropic use.

We calculate zonal statistics for a selection of some of the largest ecoregions across the dry forests and savannas (Figure S 2.2). Some of the Dinerstein et al., 2017 ecoregions are grouped together into larger units with similar biophysical and geographic space. Figure S 2.2 shows the extent of the ecoregions or ecoregion groups we calculated zonal statistics for.

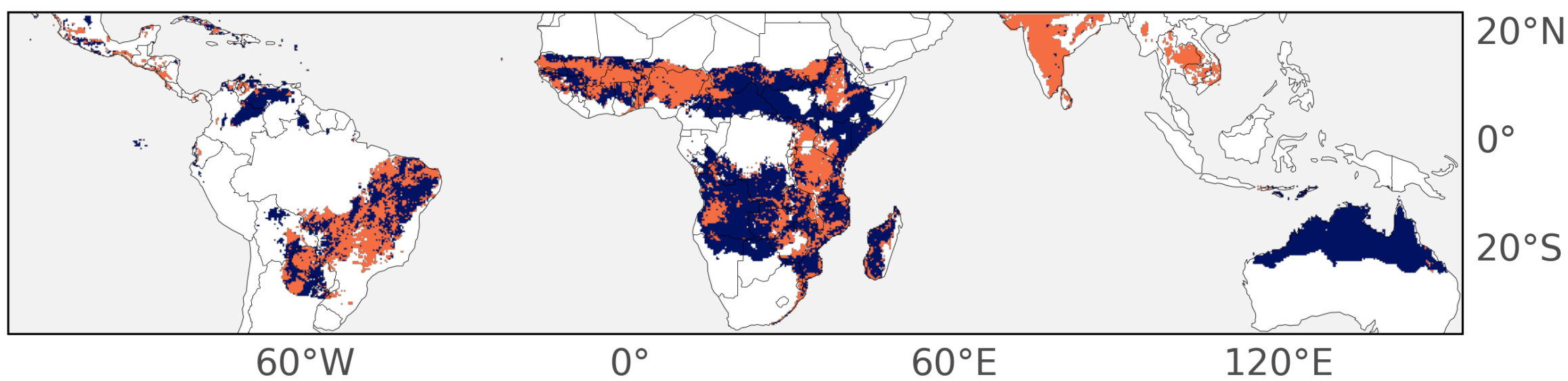

Figure S 2.1: The global extent of dry forests and savannas, classified into untransformed (dark blue) and human-transformed (orange) landscapes. The definition of transformed is based on land cover information only, and identifies landscape units mostly comprised of anthropic land covers or based on land cover maps and a tree-cover loss product. Map lines delineate study areas and do not necessarily depict accepted national boundaries.

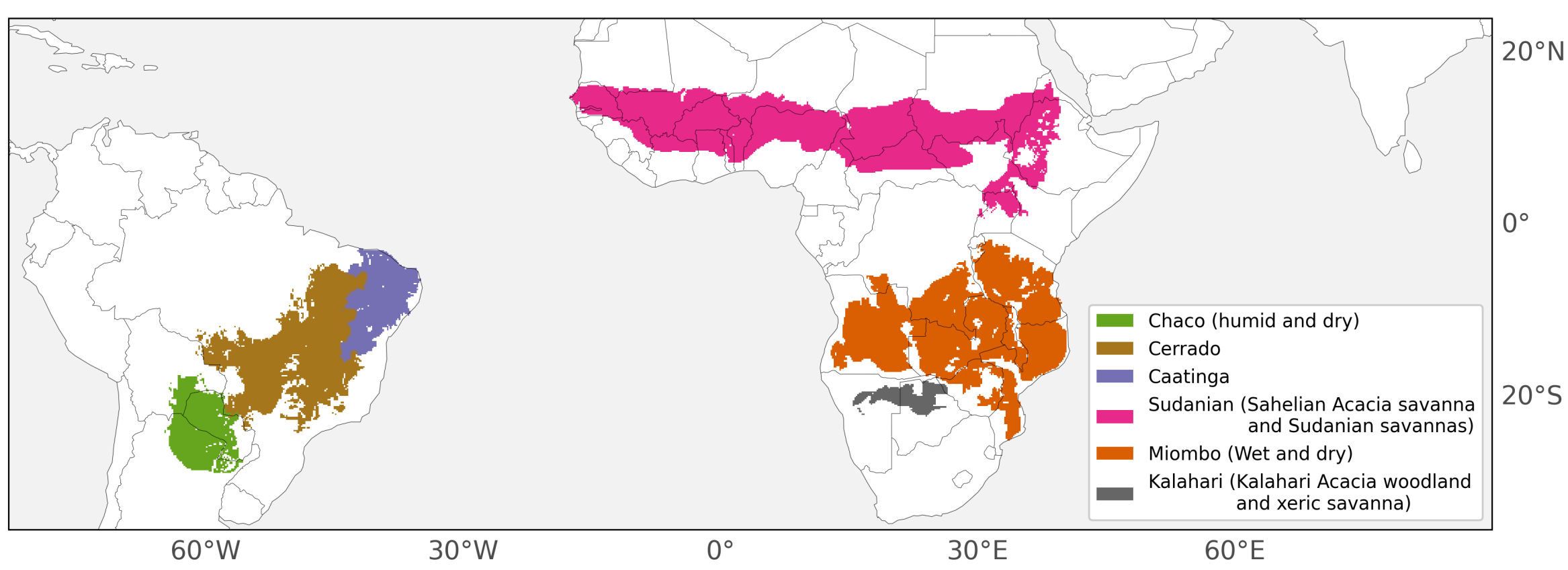

Figure S 2.2: Largest Ecoregions in the dry forests and savannas. Map lines delineate study areas and do not necessarily depict accepted national boundaries.

S 3 Plot data

We used a pan–dry-tropical plot dataset to calibrate and validate the radar–AGCD model, comprising 237 large research plots (each >0.25 ha) contributed through the SECO partnership from ForestPlots.net, SEOSAW, TERN (via the ausplotsR package), and independent collaborators. The plots span the major dry forest and savanna regions of Africa, the tropical Americas, and Asia and Australia. To align with the ALOS-2 PALSAR-2 observation record, we restricted the dataset to plot censuses conducted between 2015 and 2024 and paired each plot with co-incident annual minimum HV backscatter for the year of the census. We also filtered out plots with complicated geometries (eg. long transects) or small grouped subplots, plots on steep slopes (>20 °), and erroneously high backscatter values (>99th percentile of minimum backscatter values). Figure S 3.1 shows the distribution of the final 237 plots used in the model.

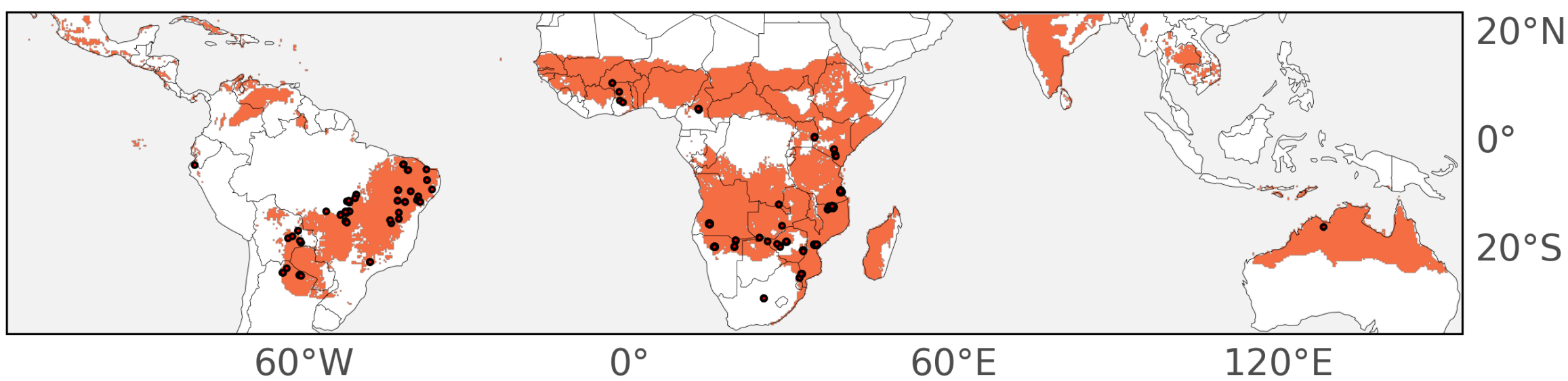

Figure S 3.1: Distribution of plots (n=237, black dots) used in the AGCD modelling across the dry forest and savanna domain (orange). Map lines delineate study areas and do not necessarily depict accepted national boundaries.

S 3.1 Details of the plot based uncertainty estimation

Minimising uncertainty in biomass measurements of a plot would require harvesting and weighing all woody vegetation within the plot for the reference data (Duncanson et al., 2021). For large datasets, this is unfeasibly resource intensive and destructive. Instead we make estimates of biomass from inventory data. Ground-based estimates of aboveground biomass density (B) of each plot (B_p) are calculated from the estimates of the biomass of all live tree stems (b) on the plot, summed and divided by the area of the plot, giving a per hectare estimate of AGBD. b is estimated based on the allometry of Chave et al., 2014, with a modified diameter-height function from Godlee et al. (in review). The allometry estimates b from measured diameter, the estimated height, and the wood density based on taxonomic identity.

Because reference plot data are not a direct measurement of biomass, but are instead an estimate (Duncanson et al., 2021), we use a Monte Carlo simulation to understand the probability distribution of B_p. This allows us to use the ground data as reference data for calibration and validation, with an understanding of its uncertainty. The Monte Carlo simulation of error follows Milodowski et al. (in prep) and includes uncertainty from the measurement of diameter in the field, the estimate of wood density, the prediction from the allometric model of height, and the allometric model used to estimate biomass (Chave et al.,

2004). A conversion factor of 0.456 was used to convert dry biomass (AGBD) to carbon equivalents (AGCD; Martin et al., 2018).

Our uncertainty estimation builds on the framework proposed by Réjou-Méchain et al. (2017). Based on field measurement experiments, Chave et al. (2004) estimated measurement error for diameter-at-breast-height (d, DBH) to be split into two categories: small errors (95%) of observations, and occasional large errors (5%). As stem-tracked repeat census data provide an opportunity for posterior filtering of large errors in d (see Godlee et al., in review), we simulate only "small" measurement error (Chave et al., 2004):

$$d' = \mathcal{N}(d, 0.0062 \cdot d + 0.0904) \qquad \text{(eq. S 1)}$$

Where d' is a random draw from a normal distribution: $\mathcal{N}(mean, sd)$.

Wood density (ρ) estimates for individual species are obtained from the global wood density database (Zanne et al., 2009), which provides a distribution of wood density estimates for many species. Uncertainties ($\sigma\rho_{species}$) are estimated following Réjou-Méchain et al. (2017), using a clipped normal distribution ensuring wood density estimates are within the globally observed range:

$$\rho' = \mathcal{N}(\rho_{species}, \sigma\rho_{species}); 0.08 \leq \rho' \leq 1.39 \qquad \text{(eq. S 2)}$$

Wood density estimates are assumed to be correlated by species. A second source of error in wood density occurs when species are misidentified and an incorrect wood density distribution is used. Errors induced by misidentification are simulated heuristically based on an assumption that misidentification occurs randomly 2% of the time. To simulate errors, species were randomly selected from the species present in the plot, weighted by abundance. This implementation ensures that species are misidentified with co-occurring species, and results in effective error rates through species misidentification that are lower in plots with low species diversity.

Height (h) for each stem was estimated based on an allometric model developed for seasonally-dry tropical systems, fitted to stem measurements of h made for 254,667 stems, across 683 dry forest and savanna plots. This follows Godlee et al. (in review), with h varying dependent on d, an environmental stress index (E; Chave et al., 2014), and plot basal area (G), but with the addition of random intercepts (u') to account for site (clusters of plots collected in the same region), and plot within site:

$$ln(h') = a' \cdot ln(d') + b \cdot E + c \cdot G' + u'_{site} + u'_{plot|site} \qquad \text{(eq. S 3)}$$

The mixed effect model above was fitted using the lme4 package (Bates et al., 2015). Uncertainties in the allometric prediction were simulated via a bootstrapping approach to predict from the posterior prediction distribution, using the simulated d' and G' to propagate measurement error.

Estimates of d', h' and ρ' were transformed into estimates of aboveground biomass (b') using a pan-tropical allometric equation (Réjou-Méchain et al., 2017):

$$ln(b') = \alpha' \cdot ln(\rho' \cdot d' \cdot h'^2) + \beta' + \epsilon' \qquad \text{(eq. S 4)}$$

Réjou-Méchain et al. (2017) published an ensemble of parameter combinations for the allometric parameters α' and β', and the resultant residual error term ϵ'. We used these parameter ensembles to simulate predictive errors in the stem-level AGB estimates for each

iteration of the Monte Carlo process. These allometric errors are treated as correlated at the species level.

We undertook 1000 iterations of the Monte Carlo procedure, resulting in 1000 stem-level estimates of AGB. These estimates are made at the individual stem level and aggregated to provide plot-level estimates of live AGBD (Mg ha^{-1}).

S 4 Contribution of plantation forestry to the dry tropical carbon budget

Based on the Global Forest Management dataset (Lesiv et al., 2022), tree plantations covered approximately 3.1 million ha across the savanna and dry forest pan-tropical extent in 2015, concentrated heavily in the Americas (2.4 million ha, 78%; table S 4.1). At the pan-tropical scale, plantations were a minor component of AGC dynamics with carbon gains and losses in plantation areas almost exactly balanced (0.0047 vs. -0.0045 Pg), leaving a net gain of 0.0002 Pg, just 0.05% of the pan-tropical net AGC change of 0.43 Pg. Plantations contributed 3.5% of total pan-tropical AGC loss from deforestation, but just 0.8% of AGC gains from densification and extensification. However, the concentration of plantations in particular regions means their local importance is much greater. Plantation rotation is a substantial component of estimated deforestation losses in the Americas and Southeast Asia, and contributes substantially to AGCD gains in Southeast Asia.

Table S 4.1: Contribution of plantation areas to pan-tropical aboveground carbon dynamics in savannas and dry forests. Areas demarcated as plantations were devided into areas with positive ΔAGCD (gains) and negative ΔAGCD (losses) based on the slope of the linear regression of time vs AGCD (2015-2024).

Continent	Area of plantation 2015 (ha)	AGC gain from extensification and densification		AGC loss from deforestation		Mean ΔAGCD in areas of gain in plantations (MgC ha-1 yr-1)	AGC gain in plantations (Pg yr-1)	Mean ΔAGCD in areas of loss in plantations (MgC ha-1 yr-1)	AGC loss in plantations (Pg yr-1)
		all land within domain (Pg yr-1)	% of which in plantations	all land within domain (Pg yr-1)	% of which in plantations				
Africa	56,454	0.4010	0.0	-0.0610	0.1	3.52	0.0001	-7.91	-0.0001
Americas	2,411,409	0.1582	2.0	-0.0503	8.3	2.96	0.0032	-3.65	-0.0042
Asia	626,640	0.0239	5.8	-0.0118	2.3	3.70	0.0014	-2.83	-0.0003
→ SEAsia	589,135	0.0101	13.6	-0.0029	7.6	3.77	0.0014	-2.96	-0.0002
→ India	37,503	0.0138	0.1	-0.0089	0.6	1.64	0.0000	-2.39	-0.0001
Australia	2,819	0.0087	0.0	-0.0038	0.0	2.15	0.0000	-1.48	0.0000
Pan-tropical	3,097,341	0.5917	0.8	-0.1269	3.5	3.15	0.0047	-3.62	-0.0045

S 5 Main GAM model description and summary

Table S 5.1: Model summary statistics for the Generalised Additive Model (GAM) fitted to aboveground woody carbon density (AGCD) using radar backscatter (HV) in the dry tropics, including estimated degrees of freedom (edf), F-statistics, and model fit.

Family: Gamma, Link function: log, Formula: AGB ~s(HV, bs = c("cr"), k = c(6))				
Parametric coefficients:				
	Estimate	Std. Error	t value	Pr(>|t|)
(Intercept)	3.48204	0.05835	59.67	<2e-16 ***
Approximate significance of smooth terms:				
	edf	Ref.df	F	p-value
s(HV)	4.123	4.626	76.61	<2e-16 ***
Signif. codes: 0 '***' 0.001 '**' 0.01 '*' 0.05 '.' 0.1 ' ' 1				
R-sq.(adj) = 0.408, Deviance explained = 56.2%, n = 237				
Approximate significance of smooth terms:				
	k'	edf	k-index	p-value
s(HV)	5.00	4.12	0.98	0.84

S 5.1 Additional validation statistics

Table S 5.2: Biomass range-specific validation statistics (Systematic deviation (eq. 2) and Root Mean Squared Deviation (RMSD; eq. 3)) following Duncanson et al. (2021)

Range of predicted AGCD values	*n* plots	RMSD	Systematic Deviation
0-10	64	5.44	-0.796
10-20	41	9.05	5.31
20-30	64	16.3	5.82
30-40	44	19	11.3
40-50	12	18.6	9.3
50+	12	30.9	24.2

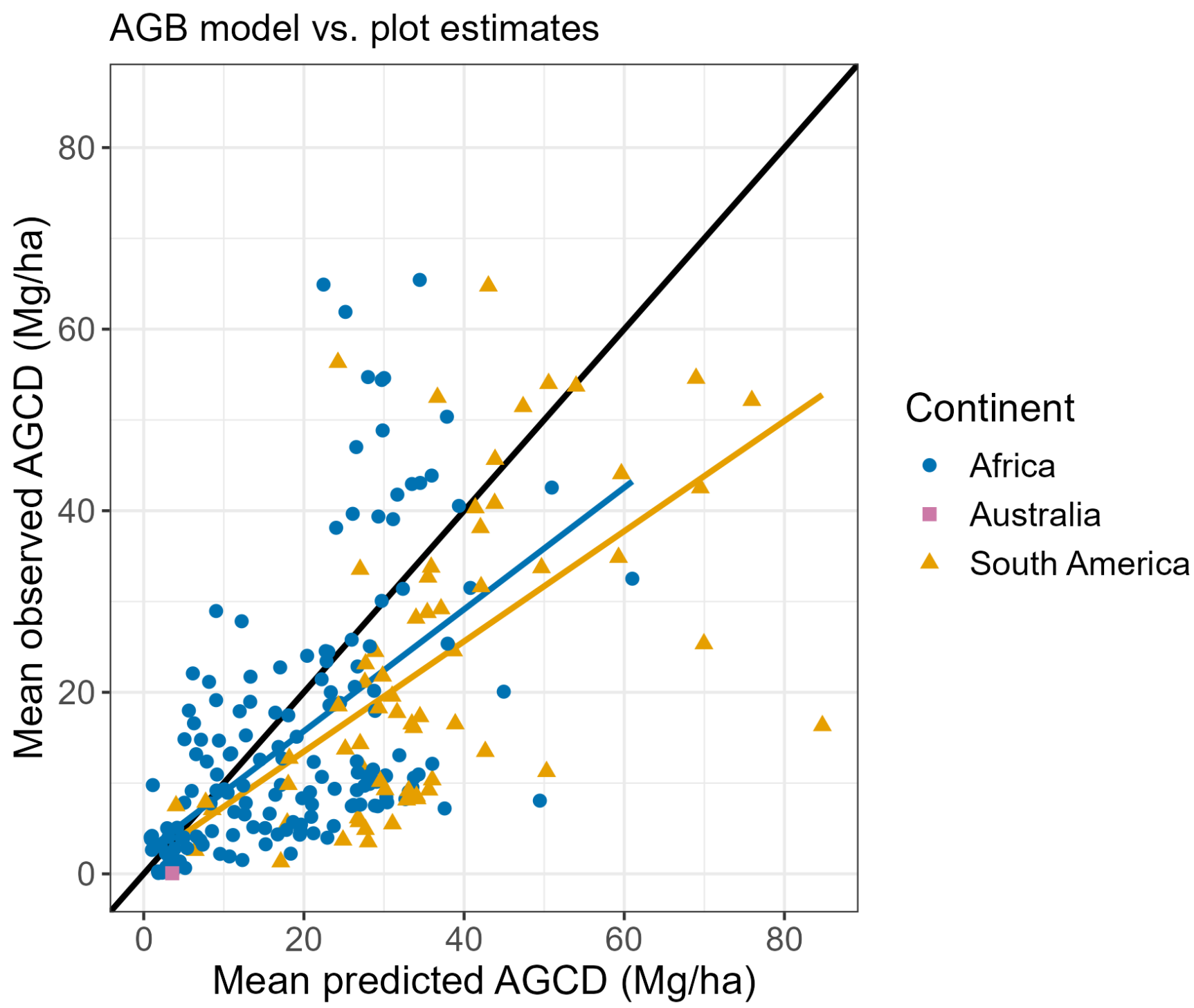

Figure S 5.1: Validation of the AGCD-backscatter model using holdout sites, grouped by continent. The black line indicates 1:1

Continent	n plots	RMSD	Systematic deviation
Africa	171	13.0	3.56
Australia	1	3.49	3.49
South America	65	19.5	12.7

Table S 5.3: Continent-specific validation statistics (Systematic deviation (eq. 2) and Root Mean Squared Deviation (RMSD; eq. 3)).

Biome	n plots	RMSD	Systematic deviation
Deserts & Xeric Shrublands	5	4.60	-3.90
Tropical & Subtropical Dry Broadleaf Forests	27	23.6	13.5
Tropical & Subtropical Grasslands, Savannas & Shrublands	188	13.7	4.90
Tropical & Subtropical Moist Broadleaf Forests	17	14.3	10.1

Table S 5.4: Biome-specific validation statistics (Systematic deviation (eq. 2) and Root Mean Squared Deviation (RMSD; eq. 3))

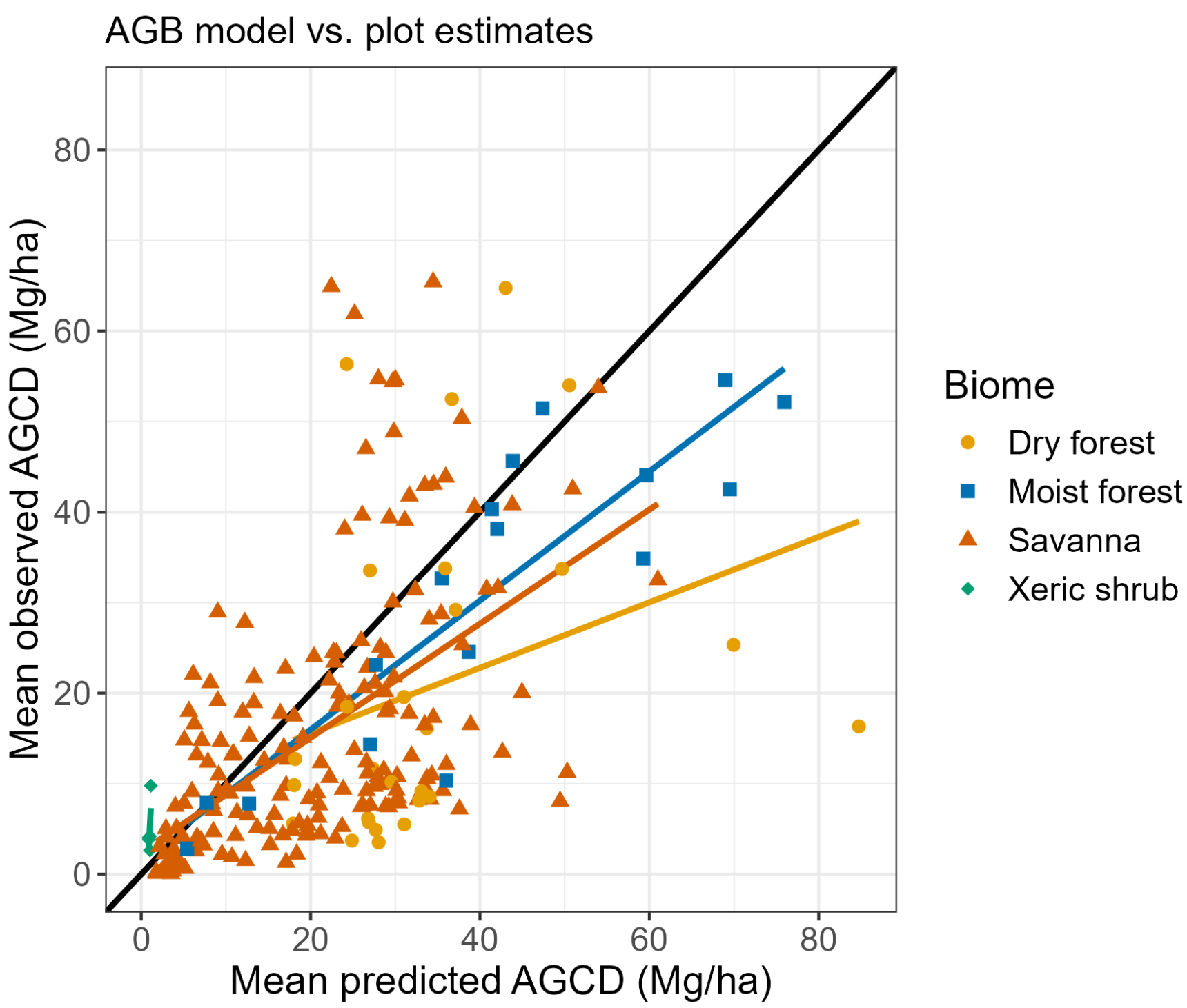

Figure S 5.2: Validation of the AGCD-backscatter model using holdout sites, grouped by biome. The black line indicates 1:1

S 5.2 Uncertainty in model

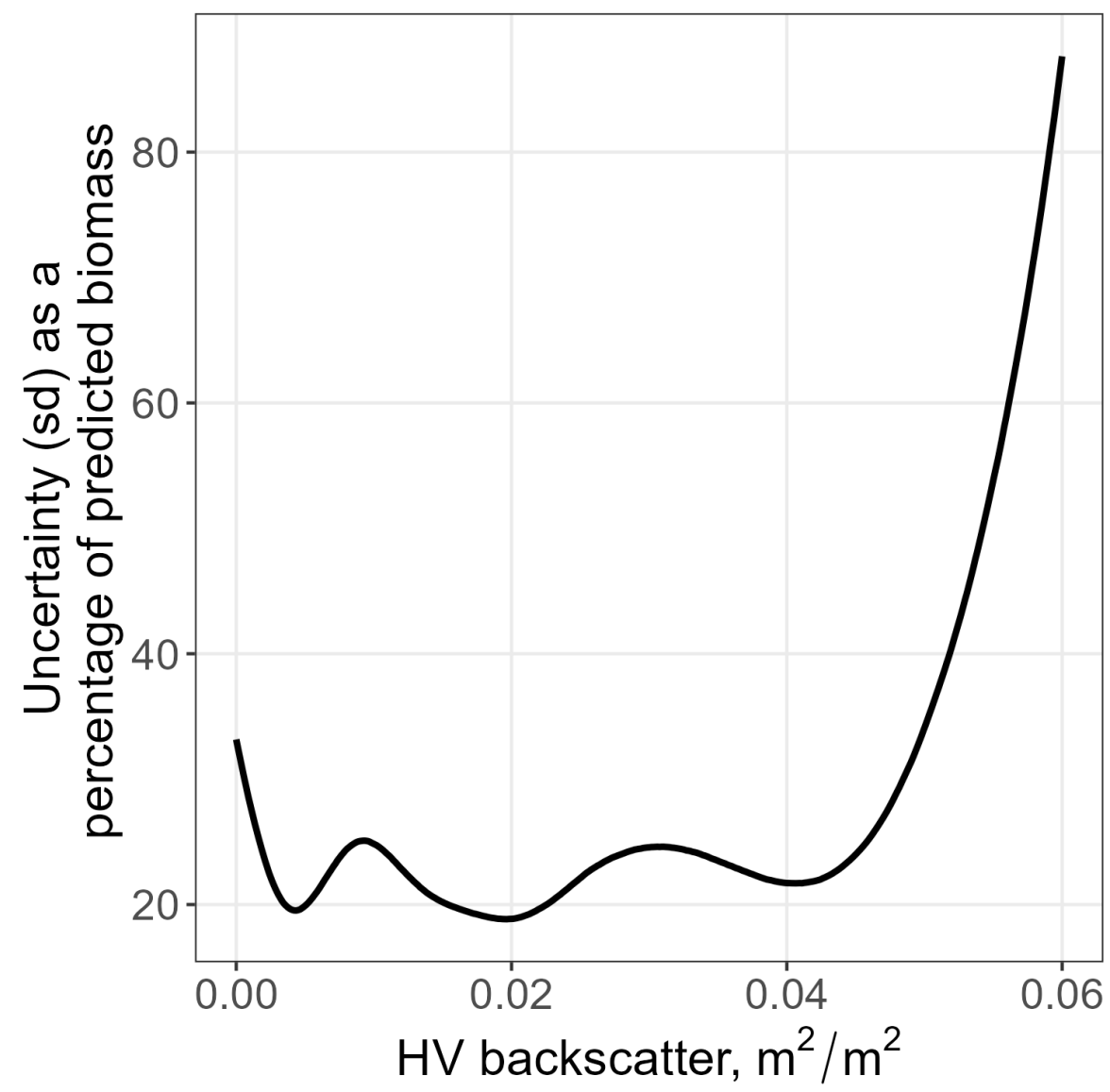

Figure S 5.3: Uncertainty in the biomass-backscatter relationship across the range of radar data observed in the training/testing data. Uncertainty is estimated with an end-to-end Monte Carlo procedure that propagates three error sources: uncertainty in plot-based AGCD (from biometry, allometry, wood density), uncertainty in the annual minimum backscatter (modelled as a normal distribution with σ =~12% of the mean, estimated from overlapping ScanSAR scenes), and statistical uncertainty in the GAM that links backscatter to AGCD

S 6 Comparison to CCI-Biomass version 6

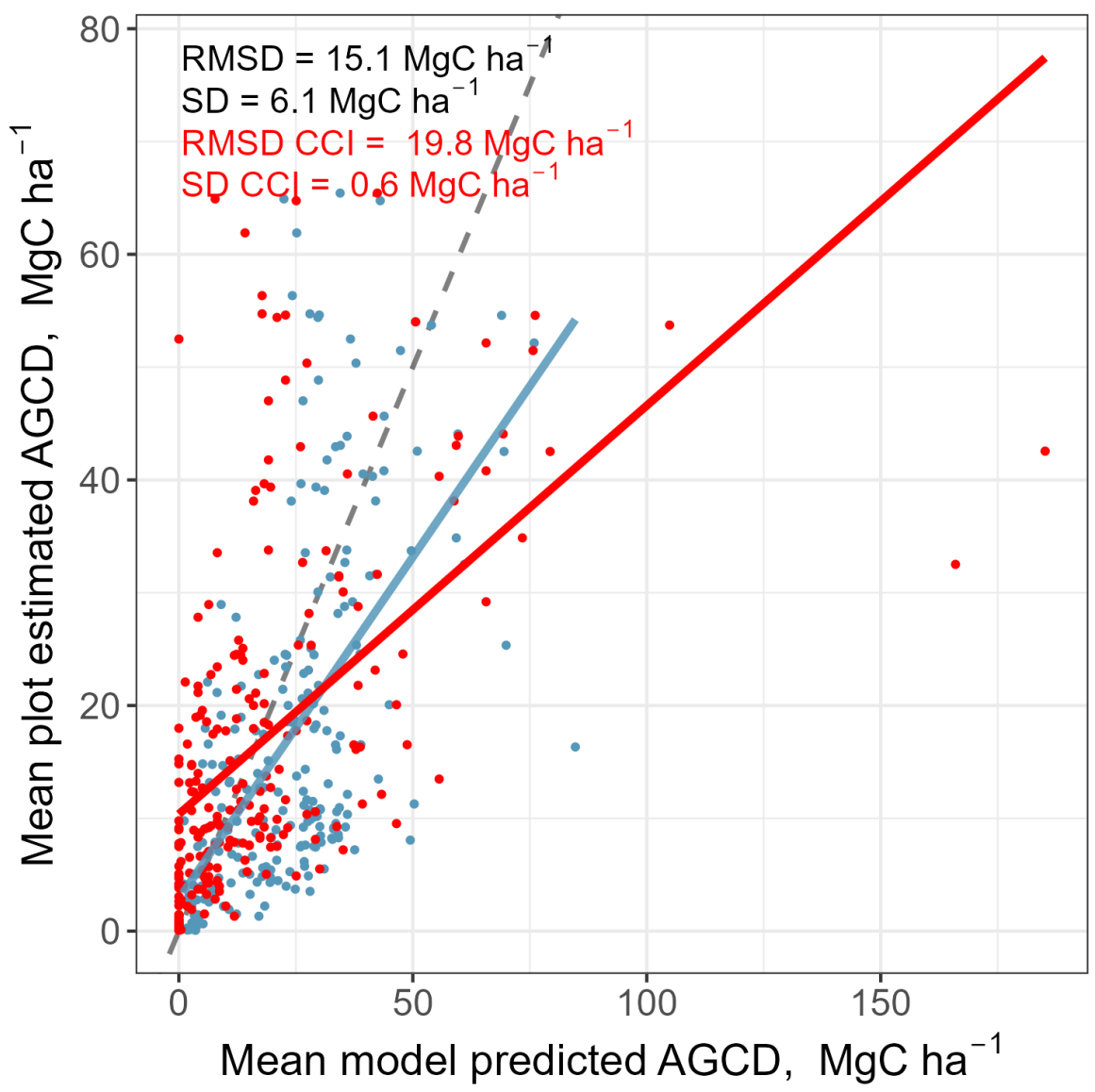

Figure S 6.1: Validation of the AGCD-backscatter model using holdout sites, and the validation of the CCI-Biomass v6 data product using the same plots. The dashed line indicates 1:1; the blue line shows the linear fit of our model, and the red line shows the CCI-Biomass linear fit. RMSD = Root Mean Squared Deviation. SD = Systematic Deviation

Table S 6.1: Biomass range-specific validation statistics (Systematic deviation (eq. 2) and Root Mean Squared Deviation (RMSD; eq. 3)) for the CCI-Biomass v6 data product against the plot data used in our GAM model, following Duncanson et al. (2021)

Range of predicted AGCD values	CCI n plots	CCI RMSD	CCI Systematic Deviation
0–10	64	7.36	-4.47
10–20	41	8.51	-2.43
20–30	64	16.3	-5.27
30–40	44	19.9	5.23
40–50	12	22.5	10.3
50+	12	62.0	42.7

Spatially, there are some areas of large differences between our estimate of AGCD and the biomass estimates from existing global products. Most notably, in the Horn of Africa and Caatinga we estimate a much higher AGCD than CCI-Biomass. We also estimate a much higher biomass density across coastal East Africa and the southern edge of our domain in Africa. Conversely, CCI-Biomass estimates higher biomass in the south of the Congo Basin, and in the north of South America. In most other ecoregions, our estimates on mean AGCD are within ± 5 MgC ha^{-1}.

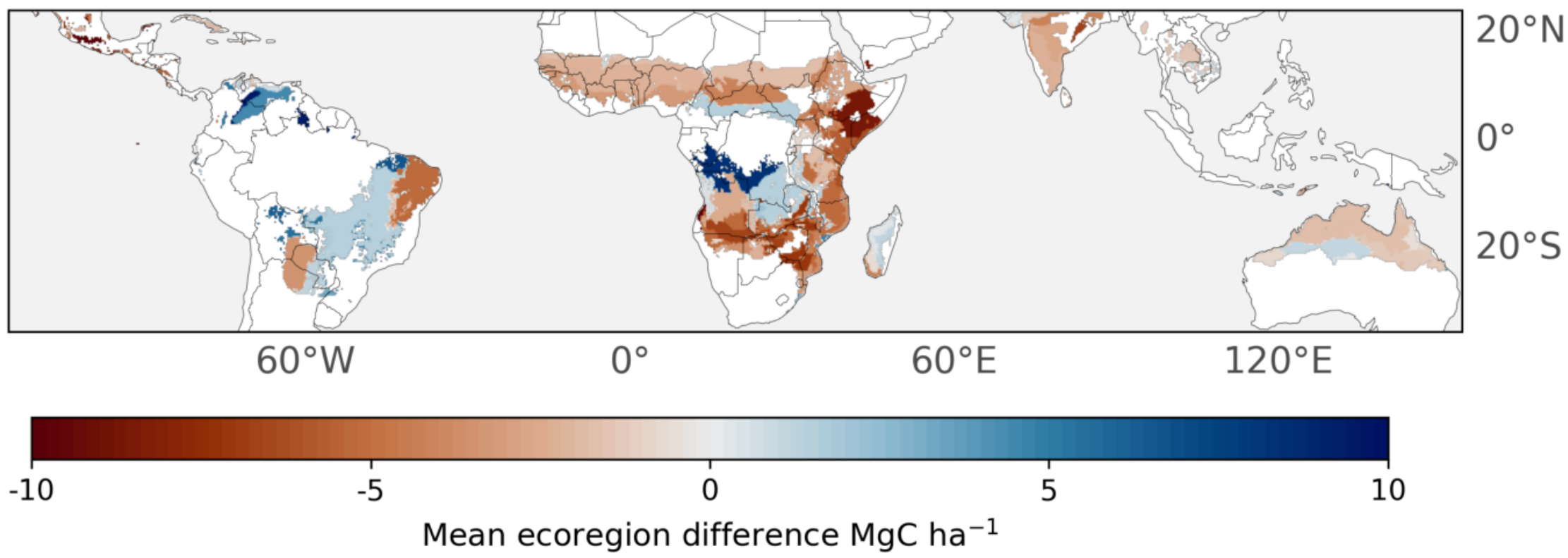

Figure S 6.2: Spatial comparison of our estimates of mean AGCD with the CCI-Biomass product (version 6) by Ecoregion (Dinerstein et al., 2017). Positive values (blue) indicate where CCI-Biomass estimates higher mean ecoregion AGCD than this study

S 7 AGCD gap-filling and temporal smoothing

We gap-filled missing and masked pixel-year AGCD estimates with a linear interpolation between the previous and next value. Where linear interpolation was not possible at the ends of time series, the missing values were back-filled/front-filled from repeating succeeding or preceding values. After gap-filling, we interpolated the time series to smooth out unrealistic values. From validation experiments, the best temporal smoothing method was a Savitzky–Golay filter with a second order polynomial. Given our small time series, we use a 5-year window for smoothing operations. To get predictions at the start and end of our time series, we pad the time series by 2 years either side using the end values. This approach ensures that each prediction, including those at the boundaries, is based on a temporal context. We present these temporally smoothed annual maps, and use the decadal mean for zonal statistics.

This smoothing approach, while improving overall prediction accuracy, involves a trade-off against sensitivity to short-term biomass changes. A five-year window will tend to remove or mask small-magnitude or short-term fluctuations, much of which may be noise, but some may be genuine disturbance and recovery events that occur within our smoothing window.

S 8 Gap-filling radar data

Where pixel backscatter values exceeded the saturation point, their AGCD is predicted at the saturation point. In total, 0.5% of the tropical dry forests and savannas domain exceeded the saturation point in at least one year. This means our estimates of the carbon stocks may be somewhat conservative.

There was an absence of ScanSAR data over much of the Australian dry forests and savannas extent for most years, with few or no images in each year. In order to ensure coverage of the full extent of tropical dry forests and savannas, we used the ALOS PALSAR-2 mosaic data as a second source of information. The PALSAR mosaics have been used for biomass mapping before (Bouvet et al., 2018; Hernández-Stefanoni et al., 2020; McNicol et al., 2018), but can suffer from striping data artefacts in many parts of the world, and may be more prone to soil moisture effects (McNicol et al., 2018). We carried out the same data extraction, GAM modelling, validation and prediction using the mosaic data to produce a second biomass map to use for Australia, and to gap-fill missing data elsewhere. The gap-filled area makes up 20% of the domain, mostly from coverage of Australia (Figure S 8.1). While the model produces less accurate results (see below) it allows us to produce a wall-to-wall pan-tropical biomass map.

Some of the change mapping using the PALSAR mosaic data have issues due to outliers in some years or certain strips of data. We used outlier detection (z score across the time series of biomass >2) to remove outlier pixels in the time series. We then carried out the same temporal smoothing as used with the ScanSAR data to produce the final biomass map from this dataset to gap-fill the maps. Uncertainty from the ALOS mosaic model is not quantified.

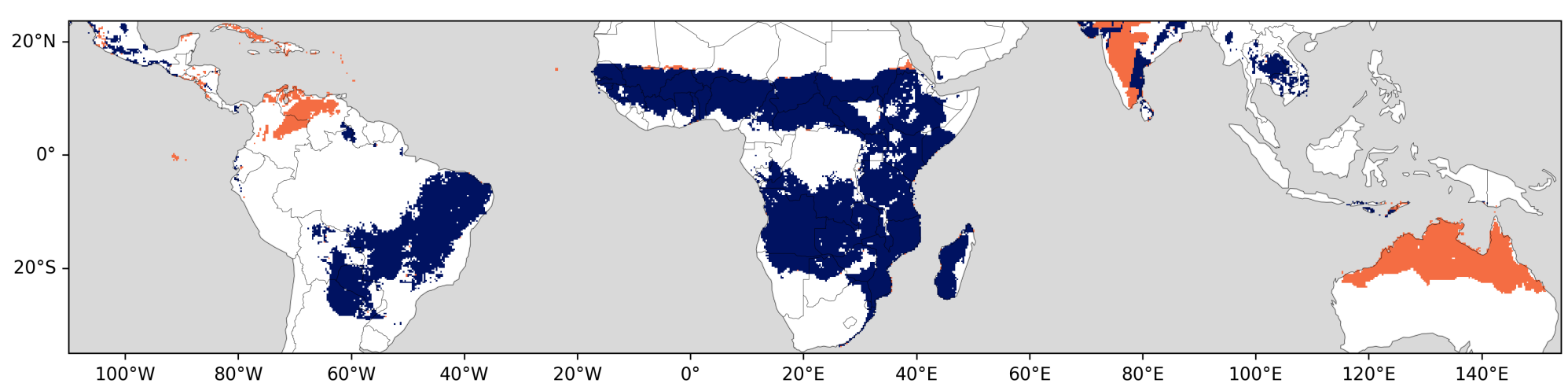

Figure S 8.1: Map showing areas where biomass is predicted from ScanSAR data (blue) and where it is gap-filled using the mosaic model (orange). Map lines delineate study areas and do not necessarily depict accepted national boundaries.

The GAM model for the PALSAR mosaic model used for gap-filling was similar to the main GAM, using the formula AGCD ~s(HV, bs = c("cr"), k = c(10)) with a Gamma distribution and a log link function. The model fit is shown in S 8.1. The model had R-sq.(adj) = 0.285; deviance explained = 45.6% ; n = 516.

Table S 8.1: Summary statistics for the mosaic model used to gap-fill. The Generalised Additive Model (GAM) is fitted to aboveground woody carbon density (AGCD) from ALOS-2 PALSAR-2 annual mosaic radar backscatter as a predictor variable in the dry tropics.

Parametric coefficients:

	Estimate	Std. Error	t value	Pr(>|t|)
(Intercept)	3.73326	0.06104	61.16	<2e-16 ***
Signif. codes: 0 '***' 0.001 '**' 0.01 '*' 0.05 '.' 0.1 ' ' 1				
Approximate significance of smooth terms:				
	edf	Ref.df	F	p-value
s(HV)	8.017	8.659	38.47	<2e-16 ***
R-sq.(adj) = 0.285, Deviance explained = 45.6% , Scale est. = 1.6444 , n = 516				
Approximate significance of smooth terms:				
	k'	edf	k-index	p-value
s(HV)	9	8.02	0.9	0.42

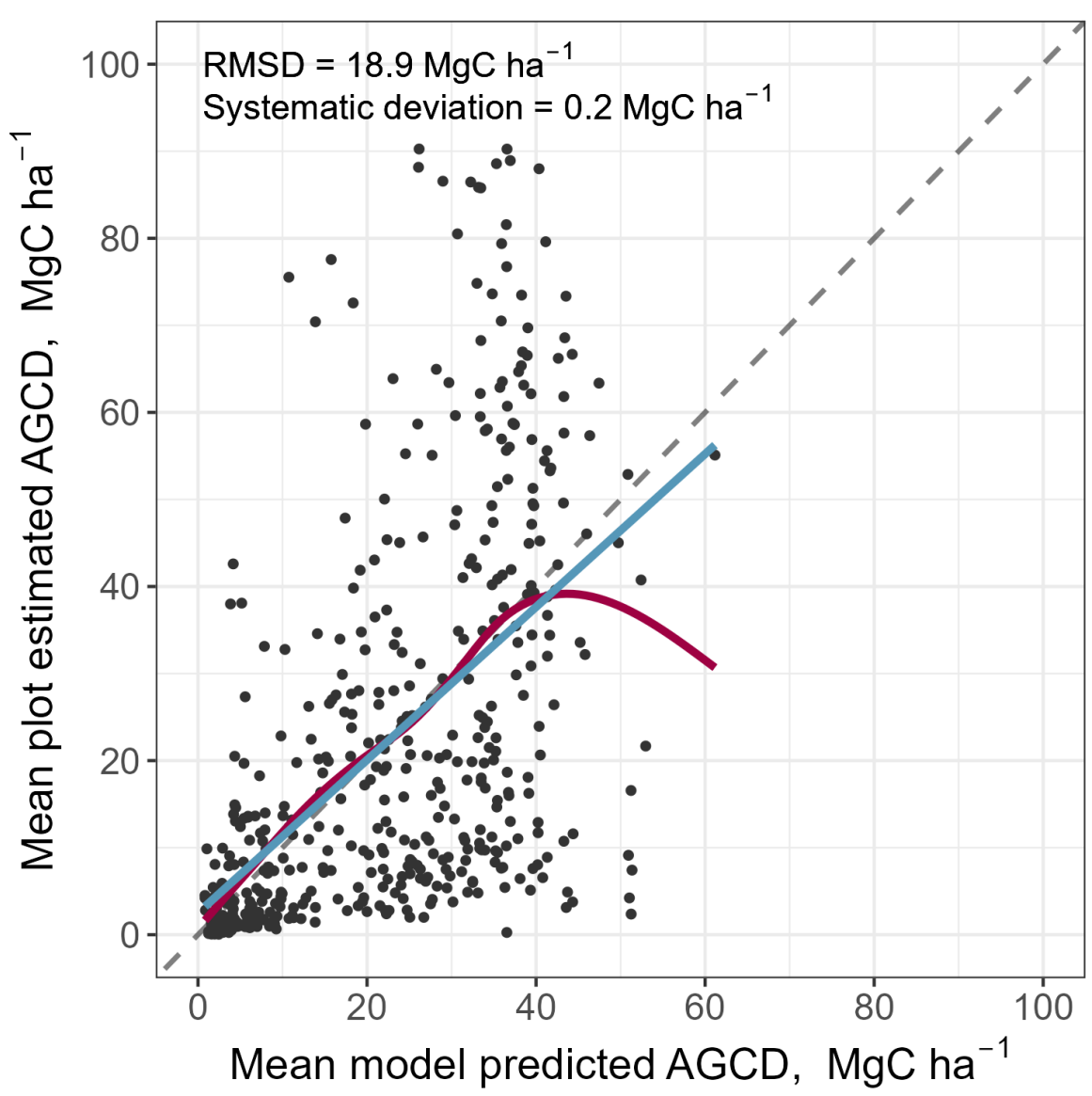

Figure S 8.2: The GAM mosaic model was validated in the same was as the main model. Validation of the AGCD-mosaic backscatter model using holdout sites. Points show the field-estimated AGCD and the mosaic model prediction. The dashed line indicates 1:1; the thick red line a GAM smooth and the blue line the linear fit. RMSD = Root Mean Squared Deviation. The model predictions are based on a leave-one-site-out procedure.

S 9 Supplementary data objects

Supplementary data objects can be found on following repository: https://doi.org/10.5281/zenodo.22877863

Supplementary data objects include:

- The plot data sheet that includes the mean plot AGCD values, standard deviation of the plot AGCD values from the monte carlo simulations, and the annual minimun HV radar backscatter in natural units for the census year from ALOS-2 PALSAR-2 ScanSAR mode for the plot data used in the model.
- The underlying data needed to create figure 3. It contains TIF files of time-averaged AGCD and AGCD change.
- The underlying data needed to create figure 6. It contains TIF files of proportion of land cover change processes at 10 km resolution.
- The pixel aboveground woody biomass density (AGBD) for each year before any masking, temporal smoothing, spatial gap filling or any transformations.
- The data frame of zonal summary statistics for continents, national boundaries and first administrative divisions. The boundary geometries used come from FAO GAUL 500m Simplified: Global Administrative Unit Layers 2015, Country Boundaries and First-Level Administrative Units. The tables present the zonal statistics of the AGC dynamics for the full zonal area within our domain and broken down by transformed / untransformed status. The AGCD change associated with each of the land cover change processes is presented alongside the area affected by each land cover process. Values shown are the totals for the zonal in our domain, averaged over the time series (2015–2024), and the area affected by each land cover change summed over the time series. Uncertainties on stocks are likely $<8\%$ of the totals and 17% of the changes.
